# Supplementary material for: Text-mined fossil biodiversity dynamics using machine learning
Source: Proc Biol Sci. 2019 Apr 24;286(1901):20190022. doi: 10.1098/rspb.2019.0022 (PMC6501925; doi:10.1098/rspb.2019.0022)
Supplement: Extended methods [file rspb20190022supp1.pdf]

1 Text-mined fossil biodiversity dynamics using machine learning: Extended methods

1.1 Plain text preprocessing

We simplified the information retrieval task by using the language detection method of Shuyo (2014) to filter any non-English documents. We attempted to remove the reference lists by doing a reverse, single match, case-insensitive regular expression search of the phrases “references”, “literature list”, and “bibliography”, and disregarding the trailing plain text. While titles of publications may contain valuable fossil occurrence information, information retrieval using the reference list could lead to improper crediting of candidate sources.

1.2 Named-entity recognition

We present simplified examples of how we performed named-entity recognition using TokensRegex expressions (Chang and Manning 2014).

```
( [/Bugula|Steginoporella/] [/cf.|aff./]{0,1} [/magnifica|bugula/]{0,1} [/sp./]{0,1} [/nov./]{0,1} )
```

The expression above will match e.g. “*Bugula*”, “*Bugula bugula*”, “*Steginoporella* cf. *magnifica*”, “*Steginoporella magnifica* sp. nov.”. Not all combinations of genus names and species epithets are valid bryozoan species names. However, we argue that this is not a problem; non-existent species such as “*Steginoporella bugula*” are not usually found in the corpus. Making this assumption when designing the rules allows the computation to be faster and more memory-efficient.

```
( [/[A-Z][.]/] [/cf.|aff./]{0,1} [/magnifica|bugula/] [/sp./]{0,1} [/nov./]{0,1} )
```

The expression above will match e.g. “*B. bugula*”, “*S. magnifica* sp. nov.”, but not “*S.* sp.” or “*S.* sp. nov.”. Named-entity recognition of geologic time intervals is simpler:

```
( [/lower|Lower|early|Early|middle|Middle|upper|Upper|late|Late/]{0,1} [/Cretaceous|Paleocene|Miocene/] )
```

This last expression will match “lower Miocene”, “Early Cretaceous”, “Paleocene”, etc. For the full named-entity recognition, we populated these expressions with comprehensive lists of genus names, species epithet names, and geologic time interval names (see main text for sources).

1.3 Shortest dependency path

As tree-based Stanford Basic Dependencies (with the “basicDependencies” parser, De Marneffe et al. 2006) have been found to perform better than the more general Universal

Dependencies (De Marneffe et al. 2014) for the purposes of relation classification (Nooralahzadeh and Øvrelid 2018), we opted to use the former. The relation between the two spans is often characterized using the shortest dependency path (SDP, e.g. Bunescu and Mooney 2005, Xu et al. 2015), and we use a similar approach here. We repeat the first example candidate from the main text (Di Martino and Taylor 2014 p. 54):

> Remarks – A few, small, infertile colonies of ***Setosellina* cf. *roulei*** have been found encrusting the undersides of very thin platy corals from the [late Burdigalian] and the **Serravallian**.

Bold font indicates the relevant spans, and square brackets indicate spans that are not currently under consideration. The dependency grammar for the example above is illustrated in Fig. S4. The shortest dependency path between the last token of the first span (*roulei*) and the first token of the last span (Serravallian) is:

$$\textit{roulei} \leftarrow \text{cf.} \rightarrow \text{found} \rightarrow \text{encrusting} \rightarrow \text{Burdigalian} \rightarrow \text{Serravallian},$$

in which "cf." is the root or lowest common ancestor. We note that "*Setosellina* cf. *roulei*" is separated by "colonies" in the dependency tree (Fig. S4), even though it is a single entity. The words along the SDP are the primary units of information. Since long SDPs were rare (0.5% had length > 12, see Fig. S5), we only considered candidates with SDP of length ≤ 12.

1.4 Labelled candidates

We created a dataset of labeled candidates as detailed in the main text. One thousand of these candidates were labeled by two annotators, of which 159 were disagreements. We collapsed the candidates that were annotated twice before fitting the classifier, as duplication of training data could lead to the classifier being overfitted or regularized. For the 861 agreed-upon candidates, we simply used one. For the 159 disagreements, we used a random gold label, as we had too few annotators to use majority rule or a similar approach. We decided not to leave out the disagreements, as artificially removing ambiguity may upward bias the performance metrics. In addition, we do not have good criteria for removing this ambiguity.

1.5 Word embeddings

A naive approach to represent words would be to treat each word in the vocabulary independently. A more efficient and commonly used approach is to project each word as a point in a lower-dimensional vector space (i.e. fewer dimensions than the vocabulary size). This is known as word embeddings or word vectors (e.g. Mikolov et al. 2013, Pennington

et al. 2014). Word embeddings are immensely useful because they significantly improve performance for almost any natural language processing task (Young et al. 2018). In preliminary analyses, we explored whether a set of word embeddings tailor-made for the bryozoan literature would perform better than a generic one. Specifically, we used English word embeddings based on the FastText skipgram model (Bojanowski et al. 2017), one pre-trained on the English Wikipedia corpus, and one trained on a subset of the bryozoan literature. Both sets of word embeddings were lower-cased, non-lemmatized, and 300-dimensional. We found that 88% of the word vocabulary in the labelled candidates were covered by the bryozoan-based word embeddings, and 64% were covered by the pre-trained Wikipedia-based word embeddings. FastText is special in that word morphology can be utilized to generate embeddings for words that are not in the embedding vocabulary. This feature of FastText is crucial for us not least because our corpus contains misspelled and rare words. Despite this, we were able to obtain word embeddings for the full word vocabulary in all of the candidates. Although the bryozoan-based word embeddings better covering the vocabulary in the labelled candidates, we chose to present results from the embeddings based on the more generic Wikipedia corpus, primarily for two reasons. First, the relation classification performed similarly for either set of word embeddings. Second, we argue that the metrics based on the Wikipedia-corpus embeddings are more representative of out-of-sample candidates. Thus, we can add additional documents to our pipeline, and more safely assume that the Wikipedia-based embeddings work just as well as before, and avoid having to retrain the embeddings and relation classifier.

1.6 Machine-learning classifiers

In our preliminary testing, we used Support Vector Machine (SVM, Cortes and Vapnik 1995), logistic regression and neural networks to classify our candidates. The SVM (using a radial-basis kernel function) and logistic regression were implemented using Scikit-learn (Pedregosa et al. 2011). Due to slightly unbalanced relation classes (56% positive, 44% negative in our training set), we weighed the inputs by the inverse frequency of their respective class when we fitted the SVM and the logistic regression. For the SVM we used two different features; “bag of words” along the SDP, and the “sum of word-vectors” along the SDP. Our baseline is a weighted coin toss (56% probability of being positive) random variable.

The neural network models we explored consist primarily of three layers. The first layer is an embedding layer (see previous section for word embeddings) that takes user-specified features as input. We experimented with several different features (Table S1, Fig. S1). These included words, part-of-speech labels, named-entity types, the governing dependency labels and SDP-subpath (“left”, “root”, “right”). Next, the embedded features were concatenated if applicable (Fig. S1A). The embedding layers are maps that translate the features into real-valued vectors, and we did this for all the tokens along the SDP (Fig.

S1A). The second is a bidirectional LSTM (Long Short-Term Memory recurrent neural network) layer with the hyperbolic tangent activator function. The third hidden layer used a binary softmax activation function. The softmax transformation ensures that the outputs y_0, y_1 are constrained to $y_0, y_1 \in [0, 1]$ and $y_0 + y_1 = 1$, thus obtaining a probability mass for the candidate relation classification task. The interpretation of the outputs y_0, y_1 follows from the criteria we set for our candidate labeling procedure. We interpret y_0, y_1 as probabilities that the sentence either 1) explicitly states or strongly implies, or 2) does not state that the species in question occurred in the given age, respectively.

We fitted the neural networks using the back-propagation algorithm (Rumelhart et al. 1986). The weight parameters of the network were randomly initialized (uniformly distributed), except for the word embedding layer. In training, the weights were iteratively optimized using gradient descent, where automatic differentiation (Abadi et al. 2016) was used to find the derivative of an optimization objective with respect to the weights. We chose to estimate the optimization objective using binary cross-entropy,

$$-(g_0 \log(y_0) + g_1 \log(y_1)),$$

where $g_0, g_1 \in \{0, 1\}$ are the gold labels, and $y_0, y_1 \in [0, 1]$ are the predictions. In other words, the optimization objective (or loss function) compares the classifier predictions with the gold labels. We used the Adam optimization algorithm (Kingma and Ba 2015) to control the learning rates. One iteration of training is one "epoch" (i.e. the classifier sees the entire training dataset once), and each epoch is subdivided in multiple "batches". We used regular and recurrent dropout rates of 0.2 in the LSTM layer, meaning that only 80% (randomly selected per batch) of the nodes in the LSTM layer were used in forward- and back-propagation during the training stage. This effectively reduces overfitting and regularizes the classifier, which in turn allows for better performance on the validation set. After training for a number of epochs, we selected the weights corresponding to the model epoch with the best F1 score (the harmonic mean of the recall and precision) evaluated on the validation set.

1.7 Classifier performances

We tested several relation classifiers in various configurations in preliminary analyses (Table S1). Our aim was not to select the best classifier, but rather to evaluate the prediction errors in a few good classifiers.

Our baseline SVM with "bag of words" (65.1% F1) performed only a little better than guessing (57.4% F1), see Table S1. Word embeddings appeared to be crucial in order to achieve good performance. Logistic regression with the "sum of word-vectors" along the SDP performed somewhat better than SVM with "bag of words" along the SDP. The non-linear SVM performs better than (linear) logistic regression when applied to

the same features. The LSTM models with domain-specific (i.e. Bryozoa) vs Wikipedia-based embeddings perform similarly, however keeping the word embedding layers static as opposed to dynamic (i.e. trainable) appears to achieve better performance. The SVM with word-vectors and the best LSTM have accuracies of 81.2% and $83.3 \pm 1.0\%$, respectively, only a little short of the inter-annotator labeling accuracy (84.1%). Several classifiers worked comparably well, and it was especially difficult to distinguish the performance between the LSTM models. As using several features did not noticeably improve performance compared with using the words along the SDP, we opted to present the simpler LSTM model in the main text.

1.8 Beginning and truncation time for geologic intervals

When we encountered a time interval preceeded by a positional or temporal adjective (e.g. "lower", "early") not found in Macrostrat, we divided the unit in three and used the appropriate part. As an example, for "Upper Messinian" we used the most recent third (5.97-5.33 Ma, million years ago) of the Messinian (7.25-5.33 Ma).

1.9 Error inspections

While most genera have reasonably short geologic durations (93% under 100 million years, 73% under 25 million years), there are some that have suspiciously long ones (Fig. S3). We used only stages and dropped (geologic) epochs, periods and eras with wider time resolution (Fig. S3). We inspected the candidates that contribute to Fig. S3, and present three exemplary problematic candidates to illustrate caveats and potential solutions. We choose to inspect false positives rather than false negatives, because the former are more problematic for the purposes of estimating diversification dynamics (see main text). The first example (from Todd et al. 1997 p. 2) is:

> "Type-species - ***Cellaria smithii*** PHILLIPS, 1829; Cornbrash (probably **Callovian**; Taylor, 1978), Scarborough, Yorkshire, England (Fig. 3.1)."

Our LSTM classifier gives a prediction of $\hat{y}_0 = 0.98$ for the relation, above the standard decision boundary of $b = 0.5$. The sentence does indeed say that *Cellaria smithii* is found in the Callovian (166.1-163.5 Ma). This extracted relation is incorrect from a taxonomic perspective, however it is not a false positive from a natural language perspective. In fact, Todd et al. (1997) argue that *Cellaria smithii* (Phillips 1829), a type species, does not belong to the genus *Cellaria*, but rather belongs to *Simplicidium*, and hence should be named *S. smithii*. As we did not account for species-level synonymy, this entry contributes to a range inflation for *Cellaria*.

A second example (from Nikulina 2002 p. 4) is:

"***Electra crustulenta*** (Pallas) The genus *Electra* Lamouroux is a member of the earliest farnily Electridae d'Orbigny known from the **Oxfordian** or [Kimmeridgian] (Pohowsky, 1973; Taylor, 1994)."

Here, the LSTM classifier gives a prediction of $\hat{y}_0 = 0.94$. Note that "*Electra crustulenta* (Pallas)" is in fact a headline, which led to incomplete sentence splitting, and the word "family" appears as "farnily" in the extracted text. Despite these faults, it is obvious to a human reader that the quote does not state that *Electra crustulenta* occurred in the Oxfordian. However, the failed sentence split is not obvious to our automatic information retrieval tools. For instance, the dependency path we used (between *Electra crustulenta* and Oxfordian) was:

$$\textit{crustulenta} \rightarrow \text{member} \rightarrow \text{d'Orbigny} \rightarrow \text{known} \rightarrow \text{Oxfordian.}$$

From the perspective of the dependency parse, it is natural to think that *Electra crustulenta* occurred in the Oxfordian, as they are connected by "member" and "known". We were not able to deduce the reference to the author "Pallas". The consequence of these errors is that the range for *Electra* is falsely inflated, apparently by more than double. The article (Nikulina 2002) appears to be translated from Russian, and it is rife with optical character recognition errors, e.g. interchanging of the letters "c" and "e", as well as "r", "t", "s" and "l", and "rn" and "m". A third example (from Ostrovsky 2013 p. 249) is:

Whereas [*Micropora*] is known from the **Cenomanian**, ***Mollia*** is much younger, having evolved in the [Danian].

Here, the classifier gives $\hat{y}_0 = 0.73$, which is less certain than for the two previous examples. This gives the wrong inference, as the sentence does not state that *Mollia* is found in the Cenomanian (100-93.9 Ma). There appears to be nothing grammatically wrong or overly ambiguous with the sentence. This type of error could have been avoided by setting a more conservative decision boundary, e.g. $b = 0.75$.

1.10 Impact of false positives on genus richness

Figure S6 shows the observed range-through genus richness as we vary the false positive rate by subsetting the dataset with various degrees of conservatism. The false positive rates (FPR) in Fig. S6 are estimated for the test dataset. However, as a large portion of candidates overlap with the training data, the net FPR is in reality smaller. As we used dropout in training (see section 1.6 Machine-learning classifiers), effectively regularizing the classifier, the FPR for the training data does not converge to zero. Interestingly, we see that the data subsets with above 0.25 FPR (at recall ≥ 0.89) result in negligible difference for the final counts.

2 References

Abadi, M., P. Barham, J. Chen, Z. Chen, A. Davis, J. Dean, M. Devin, S. Ghemawat, G. Irving, M. Isard, and others. 2016. Tensorflow: A system for large-scale machine learning. Pages 265–283 *in* 12th usenix symposium on operating systems design and implementation. The USENIX Association.

Bojanowski, P., E. Grave, A. Joulin, and T. Mikolov. 2017. Enriching word vectors with subword information. Transactions of the Association for Computational Linguistics 5:135–146.

Bunescu, R. C., and R. J. Mooney. 2005. A shortest path dependency kernel for relation extraction. Pages 724–731 *in* Proceedings of the conference on human language technology and empirical methods in natural language processing. Association for Computational Linguistics.

Chang, A. X., and C. D. Manning. 2014. TokensRegex: Defining cascaded regular expressions over tokens. Stanford University Computer Science Technical Reports 2014-02.

Cortes, C., and V. Vapnik. 1995. Support-vector networks. Machine learning 20:273–297.

De Marneffe, M.-C., T. Dozat, N. Silveira, K. Haverinen, F. Ginter, J. Nivre, and C. D. Manning. 2014. Universal stanford dependencies: A cross-linguistic typology. Pages 4585–4592 *in* Language resources evaluation conference.

De Marneffe, M.-C., B. MacCartney, and C. D. Manning. 2006. Generating typed dependency parses from phrase structure parses. Pages 449–454 *in* Proceedings of language resources evaluation conference. Genoa Italy; European Language Resources Association.

Di Martino, E., and P. D. Taylor. 2014. Miocene bryozoa from east Kalimantan, Indonesia. Part I: Cyclostomata and 'Anascan' Cheilostomata. Scripta Geologica 146:17–126.

Kingma, D. P., and J. Ba. 2015. Adam: A method for stochastic optimization. International Conference on Learning Representations.

Mikolov, T., K. Chen, G. Corrado, and J. Dean. 2013. Efficient estimation of word representations in vector space. CoRR abs/1301.3781.

Nikulina, E. 2002. The evolution of colony morphogenesis in bryozoans of the order Cheilostomata. Paleontological Journal 36:5353–5428.

Nooralahzadeh, F., and L. Øvrelid. 2018. Syntactic dependency representations in neural relation classification. arXiv:1805.11461.

Ostrovsky, A. N. 2013. Evolution of sexual reproduction in marine invertebrates: Example of gymnolaemate bryozoans. Springer.

Pedregosa, F., G. Varoquaux, A. Gramfort, V. Michel, B. Thirion, O. Grisel, M. Blondel, P. Prettenhofer, R. Weiss, V. Dubourg, J. Vanderplas, A. Passos, D. Cournapeau, M. Brucher, M. Perrot, and E. Duchesnay. 2011. Scikit-learn: Machine learning in Python. Journal of Machine Learning Research 12:2825–2830.

Pennington, J., R. Socher, and C. D. Manning. 2014. GloVe: Global vectors for word representation. Pages 1532–1543 *in* Proceedings of the 2014 conference on empirical methods in natural language processing.

Phillips, J. 1829. Illustrations of the geology of Yorkshire, or, a description of the strata and organic remains of the Yorkshire coast: Accompanied by a geological map, sections, and plates of the fossil plants and animals. York; Printed for the author by T. Wilson.

Pohowsky, R. A. 1973. A Jurassic cheilostome from England. Pages 447–461 *in* G. P. Larwood, editor. Living and Fossil Bryozoa. Academic Press, London.

Rohatgi, A. 2011. WebPlotDigitizer. https://automeris.io/WebPlotDigitizer.

Rumelhart, D. E., G. E. Hinton, and R. J. Williams. 1986. Learning representations by back-propagating errors. Nature 323:533–536.

Shuyo, N. 2014. Language detection. https://github.com/shuyo/language-detection.

Taylor, P. D. 1978. A Jurassic ctenosome bryozoan from Yorkshire. Proceedings of the Yorkshire Geological Society 42:211–216.

Taylor, P. D. 1994. An early cheilostome bryozoan from the Upper Jurassic of Yemen. Neues Jahrbuch für Geologie und Palaeontologie Abhandlungen 191:331–344.

Todd, J. A., P. Taylor, and T. Favorskaya. 1997. A bioimmured ctenostome bryozoan from the early Cretaceous of the Crimea and the new genus *Simplicidium.* Geobios 30:205–213.

Xu, Y., L. Mou, G. Li, Y. Chen, H. Peng, and Z. Jin. 2015. Classifying relations via long short term memory networks along shortest dependency paths. Pages 1785–1794 *in* Proceedings of the 2015 conference on empirical methods in natural language processing. Association for Computational Linguistics.

Young, T., D. Hazarika, S. Poria, and E. Cambria. 2018. Recent trends in deep learning based natural language processing. IEEE Computational Intelligence Magazine 13:55–75.

3 Supplementary figures

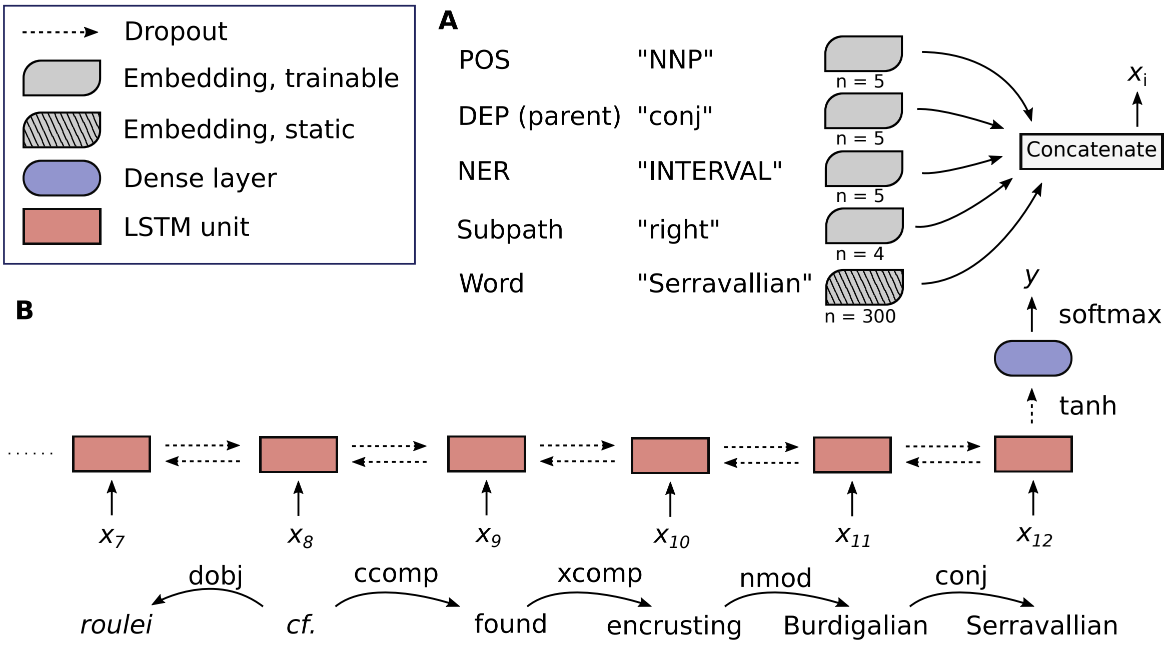

Figure S1: A: Feature embeddings. The features are part-of-speech (POS) labels, dependency labels (DEP) for the parent relationship, named-entity recognition (NER), subpath (e.g. "*roulei*" would be "left", "*cf.*" would be "root", "found" would be "right") and words into vectors of $\mathbb{R}^n$ along the shortest dependency path. The classifier discussed in the main text uses only a single feature (Word) along the SDP. **B: The unrolled architecture.** A single layer of bidirectional LSTM (45-dimensional) units in a recurrent neural network. Horizontal dashed arrows are recurrent dropout, vertical dashed arrow indicates standard dropout. The activation functions are the hyperbolic tangent (tanh) and binary softmax, $y_i = f(z_i) = e^{z_i}/(e^{z_1} + e^{z_2}), i \in \{1, 2\}$. The dependency relation types are abbreviated; dobj = direct object, ccomp = clausal complement, xcomp = open clausal complement, nmod = nominal modifier, conj = conjunct (de Marneffe et al. 2014). The figure style is inspired by Miwa and Bansal (2016).

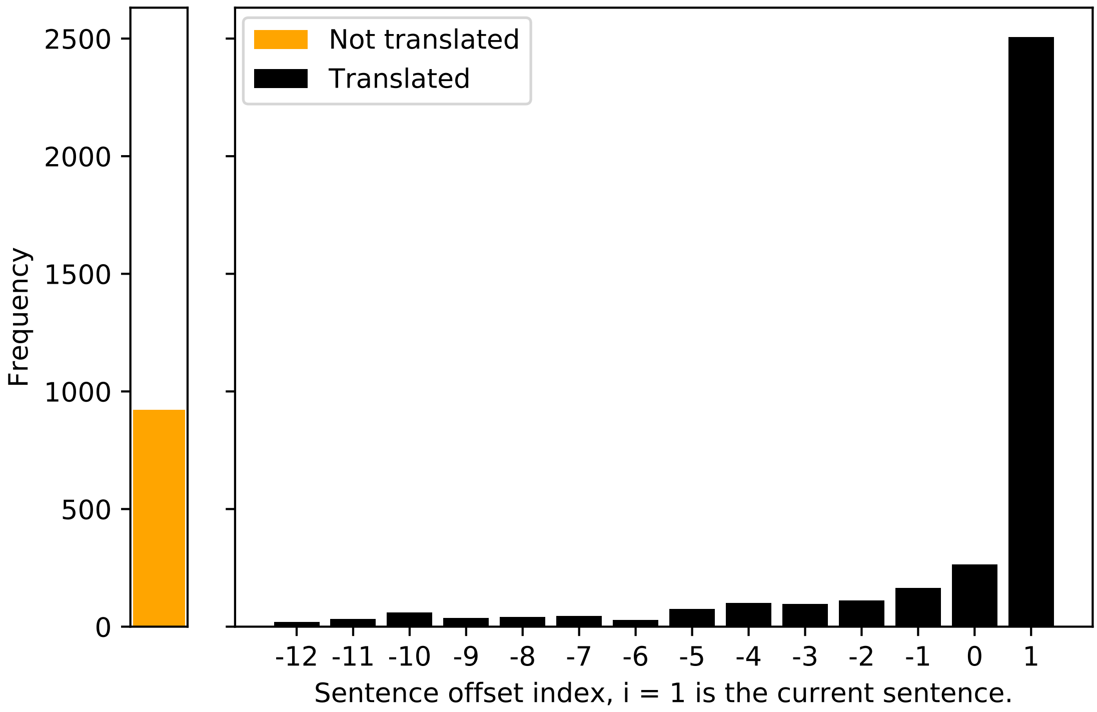

Figure S2: Simplified histogram of genus abbreviation occurrence. The majority of abbreviations (55.5%) were translated using full genus names found in the same sentence, and 24.1% abbreviations were translated using the preceeding 13 sentences. There were 4509 abbreviations in a sample of 13732 candidates. This figure does not include candidates for which the sentence exhibited two or more distinct abbreviations (1.2%), although they are also translated.

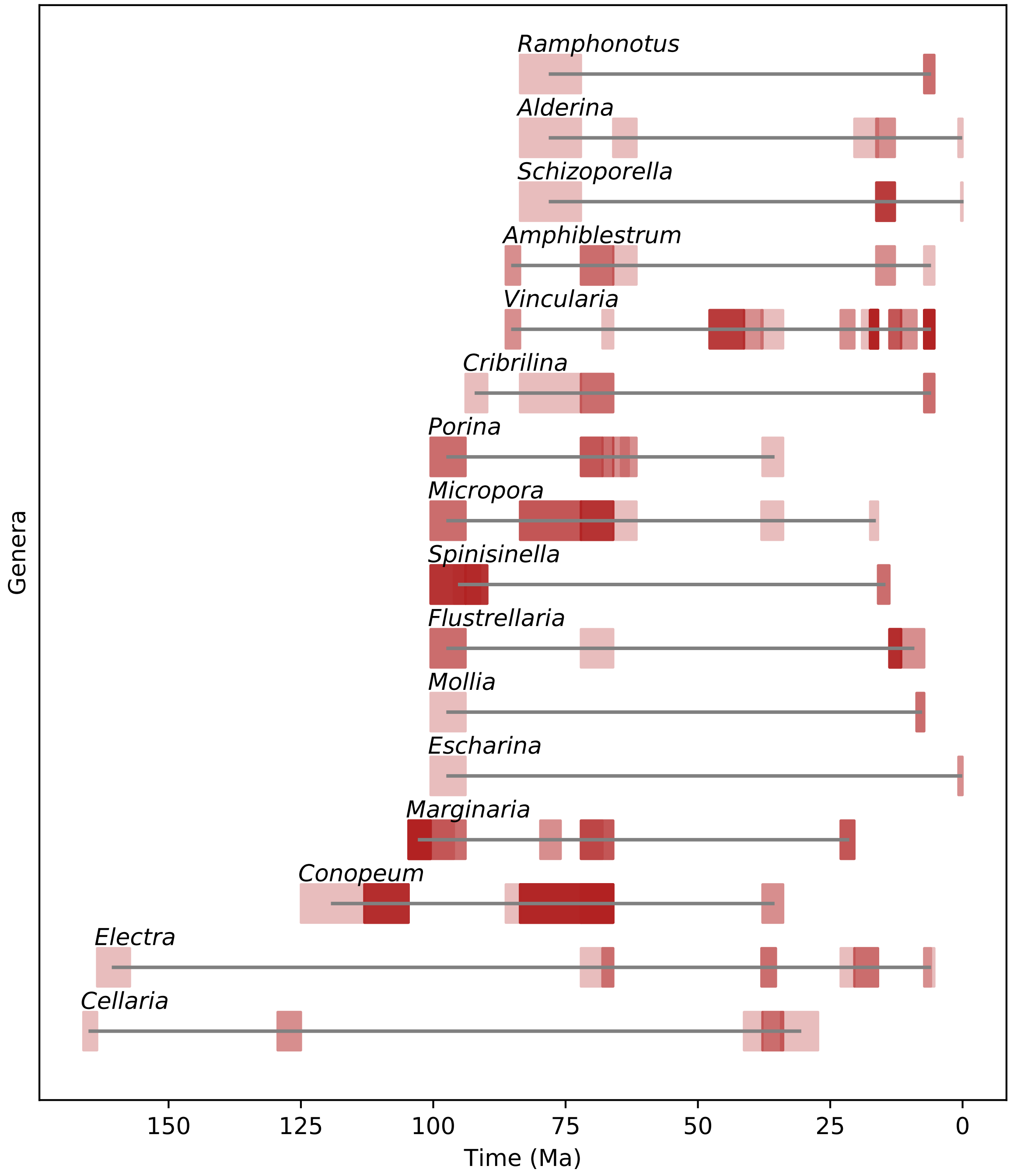

Figure S3: A selection of genera with long geologic durations. These are not supplemented with data from records of extant cheilostomes. Each rectangle is a candidate with a positive prediction, based on the neural network classifier, constrained to FPR ≤ 0.27. Transparent colour indicates a single detected candidate, stronger colours for multiple.

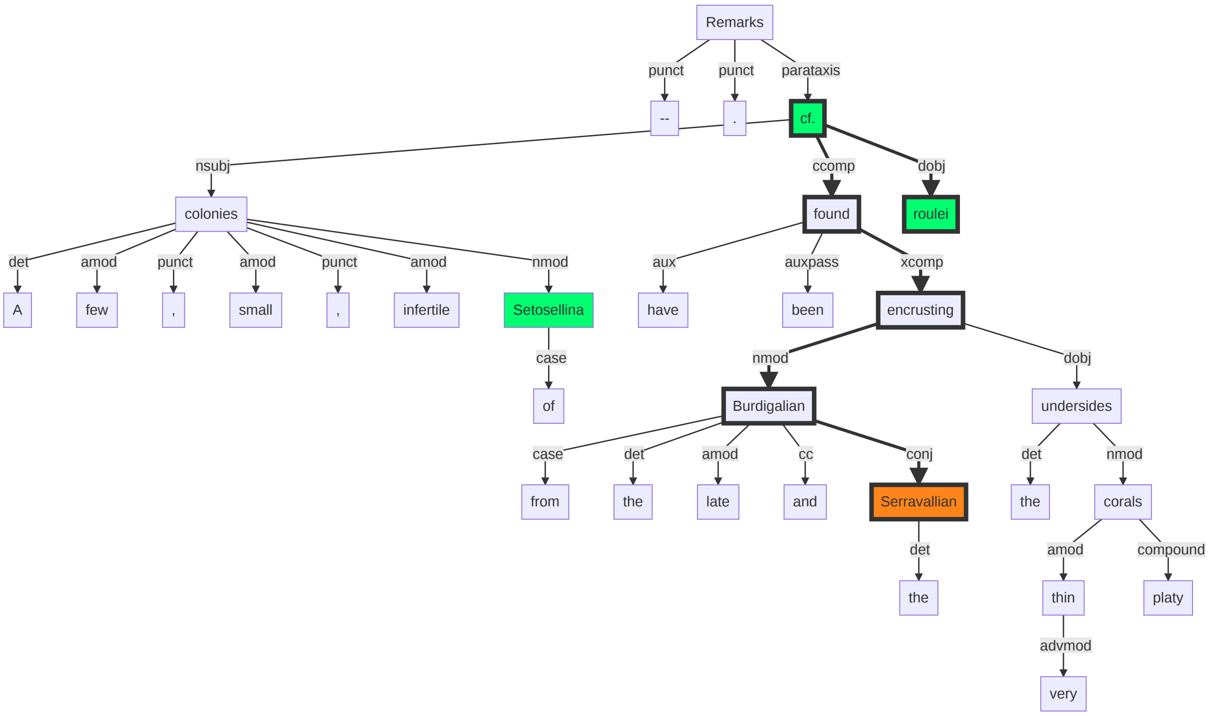

Figure S4: Dependency grammar (basic dependencies, De Marneffe et al. 2006) for first example in the main text (Di Martino and Taylor 2014). Each major box (non-grey background) is a token. Each arrow is a dependency link with a labeled dependency type (minor boxes, in grey). Bold boxes and arrows indicate the shortest dependency path between the last token (*"roulei"*) of the first span (in green, here a species) and the first token of the last span (in orange, here a geologic age).

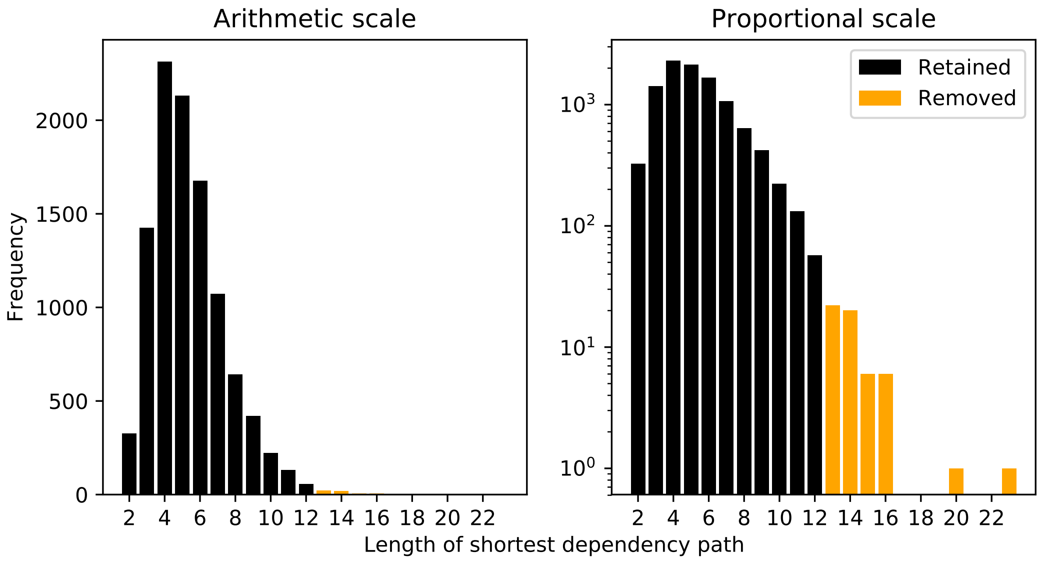

Figure S5: The distribution of labelled candidates and their shortest-dependency-path lengths. We removed candidates with a shortest dependency path of length 13 or longer. These extreme outliers represent some legitimately complicated sentences, but mostly garbled sentences that are a result of errors in optical character recognition or the tokenization procedure. The number of candidates removed from this procedure was negligible (0.5% of all candidates).

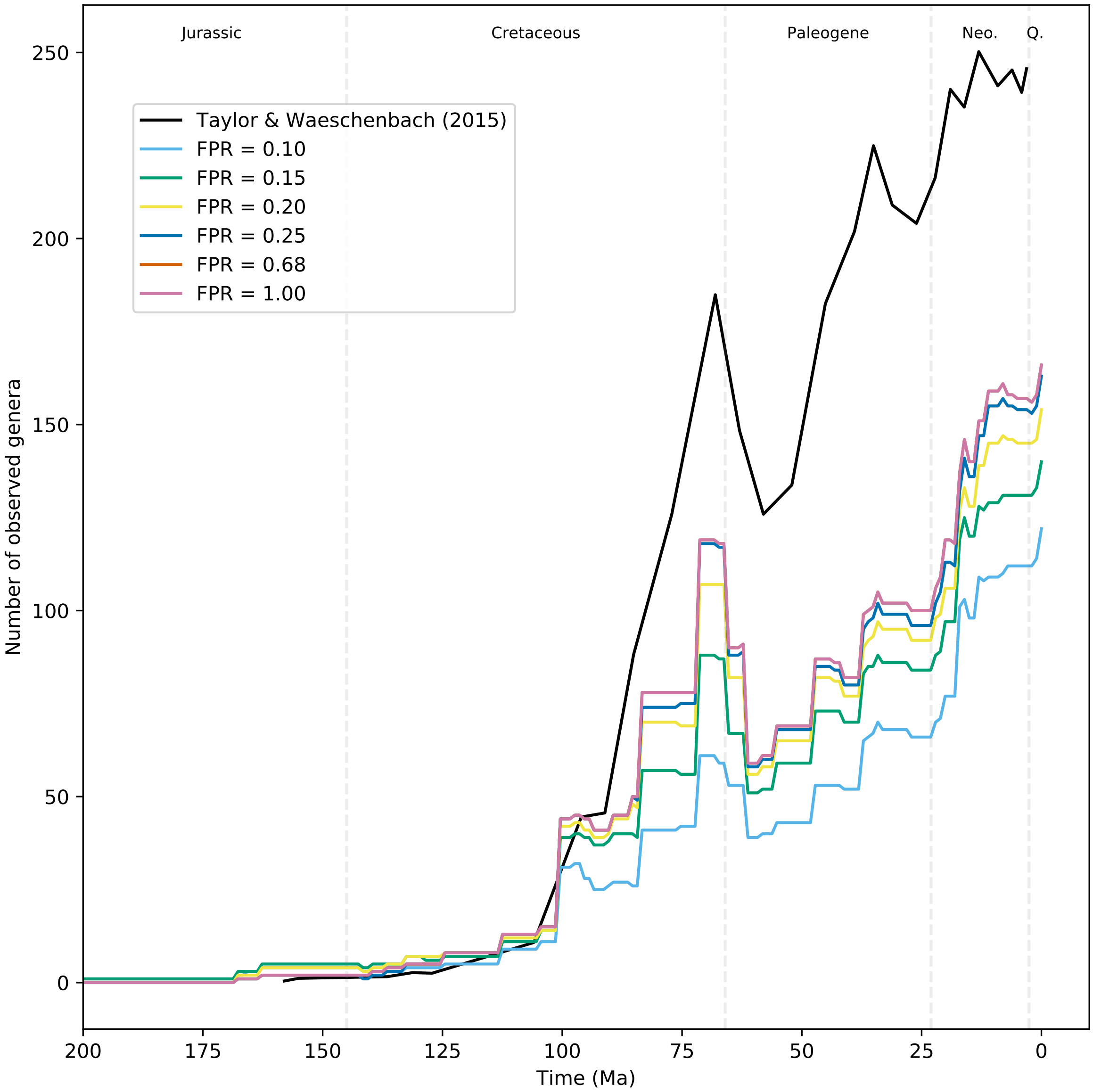

Figure S6: Range-through genus richness for cheilostomes. The curve from Taylor and Waeschenbach (2015, Fig. 12) was obtained using a plot digitizer (Rohatgi, 2011). Our richness counts are supplemented with extant observations from WoRMS (2018). The false positive rates (FPR) are estimated for the test set. The changes in range-through genus richness are small to negligible for subsets of the occurrence data with false positive rates in the interval 0.25 to 1.

4 Supplementary tables

Table S1: Summary of classifiers and features explored. The F1 is the harmonic mean of the precision and recall metrics, evaluated on the test set. SVM = Support vector machine. LSTM = Long Short-Term Memory Recurrent Neural Network. SDP = Shortest Dependency Path. The model discussed in the main text is indicated in bold.

Classifier	Features along SDP	F1 (%)		
Weighted coin	None		57.4	
SVM	Bag of words		65.1	
		Word vectors:	Wikipedia	Bryozoa
Logistic regression	Sum of word-vectors	Static	72.9	73.0
SVM	Sum of word-vectors	Static	83.9	85.3
LSTM	Words	Non-static	84.5 ± 0.5	84.2 ± 0.6
**LSTM**	**Words**	**Static**	$\mathbf{85.3 \pm 0.5}$	85.5 ± 0.4
LSTM	Words, POS, NER, DEP, Subpath	Non-static	85.1 ± 0.6	85.4 ± 0.4
LSTM	Words, POS, NER, DEP, Subpath	Static	85.9 ± 0.7	86.2 ± 0.6
