# Supplementary material for: Text-mined fossil biodiversity dynamics using machine learning
Source: Proc Biol Sci. 2019 Apr 24;286(1901):20190022. doi: 10.1098/rspb.2019.0022 (PMC6501925; doi:10.1098/rspb.2019.0022)
Supplement: Data and references [file rspb20190022supp2.pdf]

Contents

1 Cheilostome genera: range-through geologic durations 2

2 Text-mining references 11

1 Cheilostome genera: range-through geologic durations

Table S2: First and last appearances for cheilostome bryozoan genera. These are derived from the text-mined fossil occurrence data, and extant cheilostomes in the World Register of Marine Species. For each genus, the first appearance is the early boundary for the first time interval, and the last appearance is the late boundary of the last interval. Note that these first and last appearances have not been validated in any other way. Figs. 3 and S6 are based on the information given in this table. Ma = million years ago. $n = 337$.

Genus	First appearance (Ma)	Last appearance (Ma)
Acoscinopleura	83.60	72.10
Actisecos	17.46	0.00
Adeonella	8.70	0.00
Aechmella	100.50	66.00
Aeolopora	86.30	72.10
Aetea	41.30	0.00
Akatopora	86.30	0.00
Alderina	83.60	0.00
Allantopora	83.60	0.00
Amphiblestrella	72.10	68.03
Amphiblestrum	86.30	0.00
Anaptopora	100.50	93.90
Anornithopora	83.60	61.60
Anoteropora	20.44	0.00
Anotopora	100.50	93.90
Antropora	83.60	0.00
Aplousina	72.10	0.00
Arachnopusia	16.30	0.00
Arctonula	100.50	0.00
Arthropoma	23.03	0.00
Aspidostoma	83.60	0.00
Bactrellaria	83.60	72.10
Bactridium	16.30	12.80
Balantiostoma	72.10	66.00
Basslerinella	72.10	66.00
Basyaylella	11.62	7.25
Bathosella	72.10	0.00
Bathystomella	83.60	72.10
Batopora	56.00	0.00
Batrachopora	72.10	61.60
Beisselina	83.60	61.60
Beisselinopsis	72.10	61.60
Bellulopora	100.50	0.00
Biflustra	72.10	0.00
Biselenaria	41.30	37.80
Bitectipora	11.62	0.00

Table S2: First and last appearances for cheilostome bryozoan genera. These are derived from the text-mined fossil occurrence data, and extant cheilostomes in the World Register of Marine Species. For each genus, the first appearance is the early boundary for the first time interval, and the last appearance is the late boundary of the last interval. Note that these first and last appearances have not been validated in any other way. Figs. 3 and S6 are based on the information given in this table. Ma = million years ago. $n = 337$. *(continued)*

Genus	First appearance (Ma)	Last appearance (Ma)
Boreas	72.10	66.00
Boreasina	89.80	66.00
Brydonella	72.10	61.60
Bryopesanser	17.46	0.00
Buffonellaria	33.90	0.00
Bullaconopeum	75.93	66.00
Caberea	17.46	0.00
Caberoides	47.80	15.97
Calloporina	13.82	0.00
Calpidopora	100.50	93.90
Calvina	66.00	61.60
Calyptotheca	17.46	0.00
Canda	17.46	0.00
Castanopora	86.30	66.00
Catenicella	47.80	0.00
Cauloramphus	11.62	0.00
Cellaria	166.10	0.00
Cellarinidra	100.50	93.90
Celleporaria	66.00	0.00
Celleporella	72.10	0.00
Celleporina	16.30	0.00
Chaperia	12.70	0.00
Charixa	132.90	0.00
Cheethamia	83.60	66.00
Cheilopora	38.00	0.00
Cheiloporina	38.00	0.00
Chiastosella	34.30	0.00
Chiplonkarina	113.00	66.00
Cigclisula	20.44	0.00
Cleidochasmidra	0.78	0.00
Coleopora	11.62	0.00
Conopeum	125.00	0.00
Copidozoum	20.44	0.00
Corymboporella	100.50	93.90
Cosciniopsis	47.80	0.00
Coscinopleura	66.00	61.60
Costatimorpha	37.80	33.90
Cranosina	13.82	0.00
Crassimarginatella	93.90	0.00

Table S2: First and last appearances for cheilostome bryozoan genera. These are derived from the text-mined fossil occurrence data, and extant cheilostomes in the World Register of Marine Species. For each genus, the first appearance is the early boundary for the first time interval, and the last appearance is the late boundary of the last interval. Note that these first and last appearances have not been validated in any other way. Figs. 3 and S6 are based on the information given in this table. Ma = million years ago. $n = 337$. *(continued)*

Genus	First appearance (Ma)	Last appearance (Ma)
Craticulacella	83.60	75.93
Cribrilaria	56.00	0.00
Cribrilina	93.90	0.00
Criserpia	168.30	166.10
Crisidmonea	83.60	0.00
Cryptostomella	83.60	66.00
Ctenopora	100.50	93.90
Cupuladria	16.30	0.00
Cyclicopora	38.00	0.00
Cymulopora	75.93	0.00
Dacryoporella	89.80	86.30
Decurtaria	66.00	61.60
Diacanthopora	66.00	61.60
Dicasignetella	41.30	37.80
Dightonia	21.70	19.00
Dimorphomicropora	72.10	66.00
Dionella	72.10	66.00
Discoflustrellaria	47.80	41.30
Discopora	72.10	66.00
Discoporella	23.03	0.00
Discoradius	47.80	33.90
Discovibracella	66.00	61.60
Distelopora	100.50	75.93
Ditaxiporina	47.80	28.10
Dittosaria	56.00	28.10
Dysnoetocella	98.30	96.10
Ehrhardina	100.50	93.90
Ellisina	72.10	0.00
Emballotheca	8.70	0.00
Encicellaria	72.10	15.97
Escharella	72.10	0.00
Escharifora	75.93	66.00
Escharina	100.50	0.00
Escharipora	89.80	86.30
Eucratea	56.00	0.00
Euritina	100.50	89.80
Exechonella	47.80	0.00
Exochella	83.60	0.00

Table S2: First and last appearances for cheilostome bryozoan genera. These are derived from the text-mined fossil occurrence data, and extant cheilostomes in the World Register of Marine Species. For each genus, the first appearance is the early boundary for the first time interval, and the last appearance is the late boundary of the last interval. Note that these first and last appearances have not been validated in any other way. Figs. 3 and S6 are based on the information given in this table. Ma = million years ago. $n = 337$. *(continued)*

Genus	First appearance (Ma)	Last appearance (Ma)
Figularia	66.00	0.00
Filaguria	13.82	0.00
Floridina	72.10	0.00
Floridinella	72.10	0.00
Flustrellaria	100.50	0.00
Foratella	72.10	66.00
Foveolaria	93.90	0.00
Francopora	89.80	86.30
Frurionella	72.10	61.60
Fusicellaria	93.90	89.80
Gaudryanella	47.80	41.30
Gemellipora	16.30	0.00
Gemelliporella	16.30	0.00
Gephyrotes	15.97	0.00
Gigantopora	13.82	0.00
Gilbertopora	113.00	75.93
Gontarella	13.82	0.00
Graptoporella	86.30	83.60
Hagenowinella	72.10	66.00
Haplocephalopora	66.00	61.60
Haplostoechios	104.67	93.90
Hapsidopora	100.50	93.90
Hemiphylactella	20.44	15.97
Hemismittoidea	37.80	0.00
Herentia	0.26	0.00
Herpetopora	125.00	61.60
Heteractis	47.80	28.10
Heteroconopeum	100.50	61.60
Hiantopora	66.00	0.00
Hippaliosina	0.78	0.00
Hippodiplosia	20.44	2.59
Hippomenella	38.00	0.00
Hippopleurifera	16.30	0.00
Hippopodina	17.46	0.00
Hippoporella	16.30	0.00
Hippoporidra	37.80	0.00
Hippoporina	28.10	0.00
Hippothoa	100.50	0.00

Table S2: First and last appearances for cheilostome bryozoan genera. These are derived from the text-mined fossil occurrence data, and extant cheilostomes in the World Register of Marine Species. For each genus, the first appearance is the early boundary for the first time interval, and the last appearance is the late boundary of the last interval. Note that these first and last appearances have not been validated in any other way. Figs. 3 and S6 are based on the information given in this table. Ma = million years ago. $n = 337$. *(continued)*

Genus	First appearance (Ma)	Last appearance (Ma)
Hoeverella	83.60	66.00
Homalostega	83.60	61.60
Hoplitaechmella	83.60	75.93
Hoplocheilina	100.50	61.60
Houzeauina	37.80	20.44
Hybopora	100.50	93.90
Ichnopora	93.90	61.60
Inversaria	75.93	61.60
Iodictyum	21.70	0.00
Kionidella	47.80	41.30
Kleidionella	72.10	61.60
Kristerina	83.60	72.10
Kunradocella	72.10	66.00
Kylonisa	47.80	43.47
Labioporella	20.44	0.00
Lacrimula	37.80	0.00
Lagenipora	37.80	0.00
Lagynopora	72.10	68.03
Laterotecatia	68.03	66.00
Leieschara	11.62	0.00
Leptocheilopora	86.30	66.00
Lifuella	17.46	11.62
Lunulites	93.90	33.90
Macropora	59.20	0.00
Mamillopora	37.80	0.00
Margaretta	37.80	0.00
Marginaria	104.67	20.44
Melychocella	59.20	47.80
Membranipora	137.50	0.00
Membraniporidra	66.00	0.00
Metracolposa	35.27	33.90
Metrarabdotos	20.44	0.00
Metroperiella	56.00	0.00
Micropora	132.90	0.00
Microporella	17.46	0.00
Mollia	100.50	0.00
Monoceratopora	93.90	61.60
Monoporella	83.60	0.00
Multescharellina	72.10	0.00

Table S2: First and last appearances for cheilostome bryozoan genera. These are derived from the text-mined fossil occurrence data, and extant cheilostomes in the World Register of Marine Species. For each genus, the first appearance is the early boundary for the first time interval, and the last appearance is the late boundary of the last interval. Note that these first and last appearances have not been validated in any other way. Figs. 3 and S6 are based on the information given in this table. Ma = million years ago. $n = 337$. *(continued)*

Genus	First appearance (Ma)	Last appearance (Ma)
Multescharipora	83.60	72.10
Myriapora	11.62	0.00
Mystriopora	113.00	100.50
Nannopora	89.80	86.30
Nellia	72.10	0.00
Ogiva	86.30	83.60
Ogivalina	36.63	35.27
Onychocellaria	72.10	66.00
Opisthornithopora	72.10	66.00
Orbiculipora	17.46	15.97
Ornatella	100.50	93.90
Ostrovskia	11.62	7.25
Otionellina	0.34	0.00
Otopora	100.50	93.90
Oviexechonella	13.82	11.62
Pachydera	72.10	61.60
Pachydermopora	83.60	72.10
Pachythecella	68.03	61.60
Parantropora	13.82	0.00
Parasmittina	47.80	0.00
Parellisina	13.82	0.00
Parkermavella	1.63	0.00
Pavobeisselina	66.00	61.60
Pavolunulites	89.80	66.00
Peedeesella	72.10	66.00
Pelmatopora	93.90	61.60
Pentapora	0.78	0.00
Petalostegus	100.50	0.00
Petralia	23.03	0.00
Phonicosia	34.30	0.00
Phylactella	33.90	0.00
Planicellaria	66.00	15.97
Platyglena	72.10	66.00
Plesiocleidochasma	17.46	0.00
Pleurocodonellina	20.44	0.00
Pleuromucrum	20.44	0.00
Pliophloea	83.60	0.00
Porella	11.62	0.00

Table S2: First and last appearances for cheilostome bryozoan genera. These are derived from the text-mined fossil occurrence data, and extant cheilostomes in the World Register of Marine Species. For each genus, the first appearance is the early boundary for the first time interval, and the last appearance is the late boundary of the last interval. Note that these first and last appearances have not been validated in any other way. Figs. 3 and S6 are based on the information given in this table. Ma = million years ago. $n = 337$. *(continued)*

Genus	First appearance (Ma)	Last appearance (Ma)
Poricella	13.82	0.00
Poricellaria	72.10	0.00
Porina	100.50	0.00
Predanophora	17.46	11.62
Prenantia	5.33	0.00
Pseudoallantopora	72.10	66.00
Pseudothyracella	72.10	0.00
Psilosecos	63.07	61.60
Puellina	17.46	0.00
Puncturiella	72.10	66.00
Pyripora	113.00	0.00
Pyriporella	66.00	61.60
Pyriporopsis	163.50	132.90
Pyrisinella	72.10	66.00
Quadricellaria	93.90	0.00
Quasitrilaminopora	66.00	61.60
Ramicosticella	66.00	61.60
Ramphonotus	83.60	0.00
Rectonychocella	38.00	0.00
Reniporella	47.80	41.30
Reptadeonella	17.46	0.00
Reptescharella	89.80	86.30
Reptoflustrella	100.50	93.90
Reptolunulites	100.50	66.00
Reptoporina	20.44	15.97
Retelepralia	20.44	0.00
Reussirella	13.82	0.00
Rhabdopora	93.90	89.80
Rhammatopora	113.00	100.50
Rhamphostomella	56.00	0.00
Rhiniopora	83.60	66.00
Rhynchozoon	72.10	0.00
Rosseliana	34.30	0.00
Saevitella	17.46	0.00
Sandalopora	93.90	61.60
Schizomavella	20.44	0.00
Schizoporella	83.60	0.00
Schizorthosecos	35.27	33.90

Table S2: First and last appearances for cheilostome bryozoan genera. These are derived from the text-mined fossil occurrence data, and extant cheilostomes in the World Register of Marine Species. For each genus, the first appearance is the early boundary for the first time interval, and the last appearance is the late boundary of the last interval. Note that these first and last appearances have not been validated in any other way. Figs. 3 and S6 are based on the information given in this table. Ma = million years ago. $n = 337$. *(continued)*

Genus	First appearance (Ma)	Last appearance (Ma)
Schizosmittina	37.80	0.00
Schizotheca	11.62	0.00
Scorpiodinipora	23.03	0.00
Scrupocellaria	38.00	0.00
Selenaria	1.63	0.00
Semieschara	100.50	41.30
Semiescharipora	86.30	15.90
Semihaswellia	38.00	0.00
Sendinopora	13.82	11.62
Sertulipora	0.26	0.00
Setosellina	56.00	0.00
Setosinella	72.10	13.82
Smittina	17.46	0.00
Smittipora	38.00	0.00
Smittoidea	34.30	0.00
Solenonychocella	72.10	66.00
Solenophragma	83.60	66.00
Spinicharixa	125.00	100.50
Spinisinella	100.50	13.82
Stamenocella	93.90	89.80
Staurosteginopora	86.30	66.00
Steginopora	72.10	66.00
Steginoporella	47.80	0.00
Stenopsella	38.00	0.00
Stenosipora	47.80	41.30
Stephanollona	23.03	0.00
Stephanotheca	0.26	0.00
Stichocados	72.10	66.00
Stichomicropora	100.50	61.60
Stichopora	72.10	66.00
Stichoporina	47.80	41.30
Stictostega	83.60	72.10
Stylopoma	13.82	0.00
Systenostoma	72.10	68.03
Taeniocellaria	72.10	66.00
Taenioporina	72.10	66.00
Tamanicella	11.62	7.25
Tecatia	72.10	0.00
Tetraplaria	38.00	0.00

Table S2: First and last appearances for cheilostome bryozoan genera. These are derived from the text-mined fossil occurrence data, and extant cheilostomes in the World Register of Marine Species. For each genus, the first appearance is the early boundary for the first time interval, and the last appearance is the late boundary of the last interval. Note that these first and last appearances have not been validated in any other way. Figs. 3 and S6 are based on the information given in this table. Ma = million years ago. $n = 337$. *(continued)*

Genus	First appearance (Ma)	Last appearance (Ma)
Thalamoporella	47.80	0.00
Therenia	47.80	0.00
Thoracopora	100.50	93.90
Thornelya	13.82	0.00
Tornipora	72.10	61.60
Trematooecia	17.46	0.00
Tremogasterina	11.62	5.33
Tricellaria	0.26	0.00
Tricephalopora	89.80	61.60
Trichinopolia	83.60	66.00
Triphyllozoon	17.46	0.00
Triporula	16.30	0.00
Trypostega	17.46	0.00
Tubiporella	17.46	0.00
Turbicellepora	33.90	0.00
Turnerellina	72.10	66.00
Tyloporella	100.50	93.90
Ubaghsia	72.10	66.00
Umbonula	47.80	0.00
Unidistelopora	100.50	72.10
Urceolipora	21.70	0.00
Vasignyella	7.25	0.00
Vibracella	83.60	33.90
Vibracellina	56.00	0.00
Vincularia	86.30	5.33
Vix	20.44	0.00
Voorthuyseniella	23.03	20.44
Wawalia	139.80	113.00
Wilbertopora	113.00	66.00
Woodipora	72.10	66.00
Xaveropora	11.62	7.25
Zimmerella	83.60	72.10

2 Text-mining references

Abdelsalam, K. M. 2014. Benthic bryozoan fauna from the northern Egyptian coast. Egyptian Journal of Aquatic Research 40:269–282.

Adamczyk, A. D., and J. F. Pachut. 2013. Cladistic analysis of the Paleozoic bryozoan families Monticuliporidae and Mesotrypidae. Journal of Paleontology 87:635–649.

Aguirre, J., J. C. Braga, J. M. Martín, and C. Betzler. 2012. Palaeoenvironmental and stratigraphic significance of Pliocene rhodolith beds and coralline algal bioconstructions from the Carboneras Basin (SE Spain). Geodiversitas 34:115–136.

Almeida, A. C. S., F. B. C. Souza, C. M. S. Menegola, J. Sanner, and L. M. Vieira. 2014. Taxonomic review of the family Colatooeciidae Winston, 2005 (Bryozoa, Cheilostomata), with description of seven new species. Zootaxa 3868:1–61.

Almeida, A. C. S., F. B. C. Souza, J. Sanner, and L. M. Vieira. 2015. Taxonomy of recent Adeonidae (Bryozoa, Cheilostomata) from Brazil, with the description of four new species. Zootaxa 4013:348–368.

Altman, S., and R. B. Whitlatch. 2007. Effects of small-scale disturbance on invasion success in marine communities. Journal of Experimental Marine Biology and Ecology 342:15–29.

Amui, A.-M. 2005. Adeonellas from the Gulf of Aden including one new species. Zootaxa 1012:45–52.

Amui-Vedel, A.-M., P. J. Hayward, and J. S. Porter. 2007. Zooid size and growth rate of the bryozoan *Cryptosula pallasiana* Moll in relation to temperature, in culture and in its natural environment. Journal of Experimental Marine Biology and Ecology 353:1–12.

Anstey, R. L., and J. F. Pachut. 2004. Cladistic and phenetic recognition of species in the Ordovician bryozoan genus *Peronopora*. Journal of Paleontology 78:651–674.

Ariunchimeg, Y. 2012. *Admirandopora* nom. nov., a New Replacement Generic Name for a Carboniferous Bryozoan. Paleontological Journal 46:330–330.

Arnold, P. W., and P. L. Cook. 1997. Some Recent species of the genus *Anaskopora* Wass, 1975 (Bryozoa: Cribriomorpha) from Queensland. Memoirs of the Queensland Museum 42:1–11.

Álvarez, J. A. 1995. New data on the family Lichenoporidae Smitt (Bryozoa: Cyclostomida) from the Mediterranean region. Journal of Natural History 29:1067–1079.

Bader, B., and P. Schäfer. 2004. Skeletal morphogenesis and growth check lines in the Antarctic bryozoan *Melicerita obliqua*. Journal of Natural History 38:2901–2922.

Badve, R. M., and M. A. Sonar. 1995. Bryozoa Cheilostomata from Holocene, West Coast of Maharashtra, India. Geobios 28:317–335.

Banta, W. C. 1971. The body wall of cheilostome Bryozoa. IV. The frontal wall of *Schizoporella unicornis* (Johnston). Journal of Morphology 135:165–184.

Banta, W. C., N. Gray, and D. P. Gordon. 1997. A cryptocystal operculum and a new method of lophophore protrusion in the cheilostome bryozoan *Macropora levinseni*. Invertebrate Biology 116:161–170.

Banta, W. C., F. K. McKinney, and R. L. Zimmer. 1974. Bryozoan monticules: excurrent water outlets? Science 185:783–784.

Banta, W. C., F. M. Perez, and S. Santagata. 1995. A setigerous collar in *Membranipora chesapeakensis* n. sp. (Bryozoa): implications for the evolution of cheilostomes from ctenostomes. Invertebrate Biology 114:83–88.

Barnes, D. K. A. 2002. Invasions by marine life on plastic debris. Nature 416:808–809.

Barnes, D. K. A. 2006. Temporal–spatial stability of competition in marine boulder fields. Marine Ecology Progress Series 314:15–23.

Baud, A., H. A. Nakrem, B. Beauchamp, T. W. Beatty, A. F. Embry, and C. M. Henderson. 2008. Lower Triassic bryozoan beds from Ellesmere Island, High Arctic, Canada. Polar Research 27:428–440.

Benton, M. J., and P. N. Pearson. 2001. Speciation in the fossil record. Trends in Ecology & Evolution 16:405–411.

Bernecker, M., and O. Weidlich. 2005. Azooxanthellate corals in the Late Maastrichtian-Early Paleocene of

the Danish basin: bryozoan and coral mounds in a boreal shelf setting. Pages 3–25 *in* A. Freiwald and J. M. Roberts, editors. Cold-water corals and ecosystems. Springer-Verlag, Berlin.

Berning, B. 2006. The cheilostome bryozoan fauna from the Late Miocene of Niebla (Guadalquivir Basin, SW Spain): environmental and biogeographic implications. Mitteilungen aus dem Geologisch-Paläontologischen Institut der Universität Hamburg 90:7–156.

Berning, B. 2007a. Evidence for sublethal predation and regeneration among living and fossil ascophoran bryozoans. Pages 1–7 *in* S. J. Hageman, M. M. Key, and J. E. Winston, editors. Bryozoan studies 2007.

Berning, B. 2007b. The Mediterranean bryozoan *Myriapora* truncata (Pallas, 1766): a potential indicator of (palaeo-) environmental conditions. Lethaia 40:221–232.

Berning, B. 2012. Taxonomic notes on some Cheilostomata (Bryozoa) from Madeira. Zootaxa 3236:36–54.

Berning, B. 2013. New and little-known Cheilostomata (Bryozoa, Gymnolaemata) from the NE Atlantic. European Journal of Taxonomy 44:1–25.

Berning, B., and P. Kuklinski. 2008. North-east Atlantic and Mediterranean species of the genus *Buffonellaria* (Bryozoa, Cheilostomata): implications for biodiversity and biogeography. Zoological Journal of the Linnean Society 152:537–566.

Berning, B., M. Reuter, W. E. Piller, M. Harzhauser, and A. Kroh. 2009. Larger foraminifera as a substratum for encrusting bryozoans (Late Oligocene, Tethyan Seaway, Iran). Facies 55:227–241.

Berning, B., K. J. Tilbrook, and A. N. Ostrovsky. 2014. What, if anything, is a lyrula? Studi Trentini di Scienze Naturali 94:21–28.

Berning, B., K. J. Tilbrook, and A. Rosso. 2008. Revision of the north-eastern Atlantic and Mediterranean species of the genera *Herentia* and *Therenia* (Bryozoa: Cheilostomata). Journal of Natural History 42:1509–1547.

Bishop, J. D. D. 1986. The identity of *Cribrilaria innominata* (Couch, 1844) (Bryozoa, Cheilostomata). Bulletin of the British Museum (Natural History), Zoology 50:93–102.

Bishop, J. D. D. 1987. Hermit crabs associated with the bryozoan *Hippoporidra* in British waters. Bulletin of the British Museum (Natural History), Zoology 52:29–30.

Bishop, J. D. D. 1994. The genera *Cribrilina* and *Collarina* (Bryozoa, Cheilostomatida) in the British Isles and North Sea Basin, Pliocene to present day. Zoologia Scripta 23:225–249.

Bishop, J. D. D., and B. C. Househam. 1987. *Puellina* (Bryozoa; Cheilostomata; Cribrilinidae) from British and adjacent waters. Bulletin of the British Museum (Natural History), Zoology 53:1–63.

Bjerager, M., and F. Surlyk. 2007. Benthic palaeoecology of Danian deep-shelf bryozoan mounds in the Danish Basin. Palaeogeography, Palaeoclimatology, Palaeoecology 250:184–215.

Boardman, R. S. 1971. Mode of growth and functional morphology of autozooids in some Recent and Paleozoic tubular Bryozoa. Smithsonian Contributions to Paleobiology 8:1–51.

Boardman, R. S. 1984. Origin of the post-Triassic Stenolaemata (Bryozoa): a taxonomic oversight. Journal of Paleontology 58:19–39.

Boardman, R. S. 1998. Reflections on the morphology, anatomy, evolution, and classification of the class Stenolaemata (Bryozoa). Smithsonian Contributions to Paleobiology 86:1–59.

Boardman, R. S. 1999. Indications of polypides in feeding zooids and polymorphs in lower Paleozoic Trepostomata (Bryozoa). Journal of Paleontology 73:803–815.

Boardman, R. S. 2001. The growth and function of skeletal diaphragms in the colony life of lower Paleozoic Trepostomata (Bryozoa). Journal of Paleontology 75:225–240.

Boardman, R. S., and C. J. Buttler. 2005. Zooids and extrazooidal skeleton in the order Trepostomata (Bryozoa). Journal of Paleontology 79:1088–1104.

Boardman, R. S., and A. H. Cheetham. 1969. Skeletal growth, intracolony variation, and evolution in Bryozoa: a review. Journal of Paleontology 43:205–233.

Boardman, R. S., and F. K. McKinney. 1976. Skeletal architecture and preserved organs of four-sided zooids in convergent genera of Paleozoic Trepostomata (Bryozoa). Journal of Paleontology 50:25–78.

Boardman, R. S., F. K. McKinney, and P. D. Taylor. 1992. Morphology, anatomy, and systematics of the Cinctiporidae, new family (Bryozoa: Cheilostomata). Smithsonian Contributions to Paleobiology 70:1–81.

Bock, P. E., and P. L. Cook. 2001a. A review of Australian *Siphonicytara* Busk (Bryozoa: Cheilostomatida). Records of the Western Australian Museum 20:307–322.

Bock, P. E., and P. L. Cook. 2001b. Revision of the multiphased genus Corbulipora MacGillivray (Bryozoa: Cribrimorpha). Memoirs of Museum Victoria 58:191–213.

Bock, P. E., and P. L. Cook. 2002. First fossil finds of some Australian Bryozoa (Cheilostomata). Alcheringa 25:407–424.

Bock, P. E., and P. L. Cook. 2004a. Dimorphic brooding zooids in the genus *Adeona* Lamouroux from Australia (Bryozoa: Cheilostomata). Memoirs of Museum Victoria 61:129–133.

Bock, P. E., and P. L. Cook. 2004b. A review of Australian Conescharellinidae (Bryozoa: Cheilostomata). Memoirs of Museum Victoria 61:135–182.

Bone, Y., and N. P. James. 1993. Bryozoans as carbonate sediment producers on the cool-water Lacepede Shelf, southern Australia. Sedimentary Geology 86:247–271.

Bork, K. D., and T. G. Perry. 1968. Bryozoa (Ectoprocta) of Champlanian age (Middle Ordovician) from northwestern Illinois and adjacent parts of Iowa and Wisconsin. Part III. *Homotrypa, Orbignyella, Prasopora, Monticulipora*, and *Cyphotrypa*. Journal of Paleontology 42:1042–1065.

Braga, G. 2003. Salvador Reguant, Bryozoologist. Geologica Acta 1:169–171.

Braga, G., and F. Finotti. 2010. Aggiornamenti alla Bibliografia sul Phylum Bryozoa (fossili e viventi) dal 2006 al 2009 e integrazione dei lavori mancanti nella banca dati. Annali del Museo Civico, Rovereto 25:1–36.

Branch, M. L., and P. Hayward. 2005. New species of cheilostomatous Bryozoa from subantarctic Marion and Prince Edward Islands. Journal of Natural History 39:2671–2704.

Brood, K. 1976a. Cyclostomatous Bryozoa from the coastal waters of East Africa. Zoologica Scripta 5:277–300.

Brood, K. 1976b. Note on the bryozoan *Pustulopora* (*Diaperoecia*) *intricaria* Busk, 1875. Zoologia Scripta 5:231–233.

Brood, K. 1976c. Cyclostomatous Bryozoa from the Paleocene and Maestrichtian of Majunga Basin, Madagascar. Géobios 9:393–423.

Brown, D. A. 1956. Some Pliocene Polyzoa from South Australia. Annals and Magazine of Natural History, series 12 9:593–611.

Brown, G. D. 1965. Trepostomatous Bryozoa from the Logana and Jessamine limestones (Middle Ordovician) of the Kentucky Bluegrass Region. Journal of Paleontology 39:974–1006.

Burton, E. S. B. 1929. The horizons of Bryozoa (Polyzoa) in the Upper Eocene beds of Hampshire. Quarterly Journal of the Geological Society of London 85:223–241.

Busk, G. 1858. Zoophytology. On some Polyzoa collected by J.Y. Johnson, Esq., at Madeira. Quarterly Journal of Microscopical Science 6:124–130.

Busk, G. 1859. A Monograph of the Fossil Polyzoa of the Crag. Palaeontographical Society, London.

Cadée, G. C., P. J. Chimonides, and P. L. Cook. 1989. *Pseudolunularia* gen.n. (Cheilostomata), a lunulitiform bryozoan from the Indo-West Pacific. Zoologica Scripta 18:43–48.

Canu, F., and R. S. Bassler. 1919. Fossil Bryozoa from the West Indies. Publications of the Carnegie Institution 291:75–102.

Canu, F., and R. S. Bassler. 1923. North American later Tertiary and Quaternary Bryozoa. United States National Museum Bulletin 125:1–302.

Canu, F., and R. S. Bassler. 1930. The bryozoan fauna of the Galapagos Islands. Proceedings of the United States National Museum 76:1–78.

Canu, F., and G. Lecointre. 1927. Les Bryozoaires cheilostomes des Faluns de Touraine et d'Anjou. Mémoires de la Société Géologique de France, nouvelle série 3:19–50.

Carter, M. C. 2008. The functional morphology of avicularia in cheilostome bryozoans. PhD thesis, Victoria University, Wellington.

Carter, M. C., D. P. Gordon, and J. P. A. Gardner. 2010. Polymorphism and vestigiality: comparative anatomy and morphology of bryozoan avicularia. Zoomorphology 129:195–211.

Cheetham, A. H. 1962. Eocene Bryozoa from the McBean Formation in Georgia. Micropaleontology 8:323–336.

Cheetham, A. H. 1966. Cheilostomatous Polyzoa from the Upper Bracklesham Beds (Eocene) of Sussex. Bulletin of the British Museum (Natural History), Geology 13:1–115.

Cheetham, A. H. 1971. Functional morphology and biofacies distribution of cheilostome Bryozoa in the Danian Stage (Paleocene) of southern Scandinavia. Smithsonian Contributions to Paleobiology 6:1–87.

Cheetham, A. H. 1975. Preliminary report on Early Eocene cheilostome bryozoans from Site 308 - Leg 32, Deep-Sea Drilling Project. Initial Reports of the Deep Sea Drilling Project 32:835–851.

Cheetham, A. H., L. C. Hayek, and E. Thomsen. 1981. Growth models in fossil arborescent cheilostome bryozoans. Paleobiology 7:68–86.

Cheetham, A. H., J. B. C. Jackson, and J. Sanner. 2001. Evolutionary significance of sexual and asexual modes of propagation in Neogene species of the bryozoan *Metrarabdotos* in tropical America. Journal of Paleontology 75:564–577.

Cheetham, A. H., and D. M. Lorenz. 1976. A vector approach to size and shape comparisons among zooids in cheilostome bryozoans. Smithsonian Contributions to Paleobiology 29:1–55.

Cheetham, A. H., and P. A. Sandberg. 1964. Quaternary Bryozoa from Louisiana mudlumps. Journal of Paleontology 38:1013–1046.

Cheetham, A. H., J. Sanner, and J. B. C. Jackson. 2007. *Metrarabdotos* and related genera (Bryozoa: Cheilostomata) in the late Paleogene and Neogene of tropical America. Journal of Paleontology 81:1–91.

Cheetham, A. H., J. Sanner, P. D. Taylor, and A. N. Ostrovsky. 2006. Morphological differentiation of avicularia and the proliferation of species in mid-Cretaceous *Wilbertopora* Cheetham, 1954 (Bryozoa: Cheilostomata). Journal of Paleontology 80:49–71.

Cheetham, A. H., and E. Thomsen. 1981. Functional morphology of arborescent animals: strength and design of cheilostome bryozoan skeletons. Paleobiology 7:355–383.

Chimonides, P. J. 1987. Notes on some species of the genus *Amathia* (Bryozoa, Ctenostomata). Bulletin of the British Museum (Natural History), Zoology 52:307–358.

Chimonides, P. J., and P. L. Cook. 1994. Notes on the genus *Cranosina* (Bryozoa, Cheilostomida). Zoologia Scripta 23:43–49.

Clark, N., M. Williams, B. Okamura, J. Smellie, A. Nelson, T. Knowles, P. D. Taylor, M. J. Leng, J. Zalasiewicz, and A. M. Haywood. 2010. Early Pliocene Weddell Sea seasonality determined from bryozoans. Stratigraphy 7:199–206.

Cleary, D., and P. N. Wyse Jackson. 2007. *Stenophragmidium* Bassler, 1952 (Trepostomida: Bryozoa) from the Mississippian of Ireland and Britain. Irish Journal of Earth Sciences 25:1–25.

Clin, B., C. Stosse-Guevel, M.-F. Marquignon, L. Verneuil, and M. Letourneux. 2008. Professional photosensitive eczema of fishermen by contact with bryozoans: disabling occupational dermatosis. International Maritime Health 59:1–4.

Cocito, S., and F. Ferdeghini. 2000. Morphological variations in Pentapora fascialis (Cheilostomatida, Ascophorina). Pages 176–181 *in* A. Herrera Cubilla and J. B. C. Jackson, editors. Proceedings of the 11th international bryozoology association conference, 1998. Smithsonian Tropical Research Institute, Balboa, Republic of Panama.

Cook, P. L. 1964. Polyzoa from West Africa. I. Notes on the Steganoporellidae, Thalamoporellidae and Onychocellidae (Anasca, Coilostega). Annales de l'Institut Oceanographique (Calypso IV) 41:43–79.

Cook, P. L. 1965a. Polyzoa from West Africa. The Cupuladriidae (Cheilostomata, Anasca). Bulletin of the British Museum (Natural History), Zoology 13:189–227.

Cook, P. L. 1965b. Notes on the Cupuladriidae (Polyzoa, Anasca). Bulletin of the British Museum (Natural

History), Zoology 13:151–187.

Cook, P. L. 1967. Polyzoa (Bryozoa) from West Africa. The Pseudostega, the Cribrimorpha and some Ascophora. Bulletin of the British Museum (Natural History), Zoology 15:321–351.

Cook, P. L. 1982. Notes on some African Adeonellidae (Bryozoa, Cheilostomata). Journal of Natural History 16:833–846.

Cook, P. L., and P. E. Bock. 1994. The astogeny and morphology of *Rhabdozoum wilsoni* Hincks (Anasca, Buguloidea). Pages 47–50 *in* P. J. Hayward, J. S. Ryland, and P. D. Taylor, editors. Biology and palaeobiology of bryozoans. Olsen & Olsen, Fredensborg.

Cook, P. L., and P. E. Bock. 2002. Notes on astogeny of some Petraliellidae (Bryozoa) from Australia. Journal of Natural History 36:1601–1619.

Cook, P. L., and P. J. Chimonides. 1981a. Morphology and systematics of some rooted cheilostome Bryozoa. Journal of Natural History 15:97–134.

Cook, P. L., and P. J. Chimonides. 1981b. Morphology and systematics of some interior-walled cheilostome Bryozoa. Bulletin of the British Museum (Natural History), Zoology 41:53–89.

Cook, P. L., and P. J. Chimonides. 1984a. Recent and fossil Lunulitidae (Bryozoa: Cheilostomata). 1. The genus *Otionella* from New Zealand. Journal of Natural History 18:227–254.

Cook, P. L., and P. J. Chimonides. 1984b. Recent and fossil Lunulitidae (Bryozoa: Cheilostomata). 2. Species of *Helixotionella* gen. nov. from Australia. Journal of Natural History 18:255–270.

Cook, P. L., and P. J. Chimonides. 1985a. Recent and fossil Lunulitidae (Bryozoa: Cheilostomata). 3. 'Opesiulate' and other species of *Selenaria* sensu lato. Journal of Natural History 19:285–322.

Cook, P. L., and P. J. Chimonides. 1985b. Recent and fossil Lunulitidae (Bryozoa: Cheilostomata). 5. *Selenaria alata* Tenison Woods, and related species. Journal of Natural History 19:337–358.

Cook, P. L., and P. J. Chimonides. 1986. Recent and fossil Lunulitidae (Bryozoa, Cheilostomata). 6. *Lunulites* sensu lato and the genus Lunularia from Australasia. Journal of Natural History 20:681–705.

Cook, P. L., and P. J. Chimonides. 1987. Recent and fossil Lunulitidae (Bryozoa, Cheilostomata). 7. *Selenaria maculata* (Busk) and allied species from Australasia. Journal of Natural History 21:933–966.

Cook, P. L., and P. J. Hayward. 1983. Notes on the family Lekythoporidae (Bryozoa, Cheilostomata). Bulletin of the British Museum (Natural History), Zoology 45:55–76.

Cook, P. L., and R. Lagaaij. 1976. Some Tertiary and Recent conescharelliniform Bryozoa. Bulletin of the British Museum (Natural History), Zoology 29:319–376.

Crowley, S. F., and P. D. Taylor. 2000. Stable isotope composition of modern bryozoan skeletal carbonate from the Otago Shelf, New Zealand. New Zealand Journal of Marine and Freshwater Research 34:331–351.

Cuffey, R. J., R. M. Feldmann, and K. E. Pohlable. 1981. New Bryozoa from the Fox Hills Sandstone (Upper Cretaceous, Maestrichtian) of North Dakota. Journal of Paleontology 55:401–409.

Deudero, S., A. Blanco, A. Box, G. Mateu-Vicens, M. Cabanellas-Reboredo, and A. Sureda. 2010. Interaction between the invasive macroalga *Lophocladia lallemandii* and the bryozoan *Reteporella grimaldii* at seagrass meadows: density and physiological responses. Biological Invasions 12:41–52.

Di Geronimo, I., C. Messina, A. Rosso, R. Sanfilippo, F. Sciuto, and A. Vertino. 2005. Enhanced biodiversity in the deep: Early Pleistocene coral communities from southern Italy. Pages 61–86 *in* A. Freiwald and J. M. Roberts, editors. Cold-water corals and ecosystems. Springer-Verlag, Berlin.

Di Martino, E., and A. Rosso. 2015. Revision of the bryozoan genus *Gephyrotes* Norman, 1903 (Cheilostomata, Cribrilinidae) with the description of two new taxa. Zootaxa 3941:261–283.

Di Martino, E., and P. D. Taylor. 2012a. Pyrisinellidae, a new family of anascan cheilostome bryozoans. Zootaxa 3534:1–20.

Di Martino, E., and P. D. Taylor. 2012. Morphology and palaeobiogeography of *Retelepralia*, a distinctive cheilostome bryozoan new to the fossil record. Neues Jahrbuch für Geologie und Paläontologie-Abhandlungen 263:67–74.

Di Martino, E., and P. D. Taylor. 2012b. Systematics and life history of Antoniettella exigua, a new genus and species of cribrimorph bryozoan from the Miocene of East Kalimantan (Indonesia). Bollettino della Societá Paleontologica Italiana 51:99–108.

Di Martino, E., and P. D. Taylor. 2014a. A brief review of seagrass-associated bryozoans, Recent and fossil. Studi Trentini di Scienze Naturali 94:79–94.

Di Martino, E., and P. D. Taylor. 2014b. Miocene Bryozoa from East Kalimantan, Indonesia. Part I: Cyclostomata and 'Anascan' Cheilostomata. Scripta Geologica 146:17–126.

Di Martino, E., and P. D. Taylor. 2015. Miocene Bryozoa from East Kalimantan, Indonesia. Part II: 'Ascophoran' Cheilostomata. Scripta Geologica 148:1–142.

Dick, M. H., A. V. Grischenko, and S. F. Mawatari. 2005. Intertidal Bryozoa (Cheilostomata) of Ketchikan, Alaska. Journal of Natural History 39:3687–3784.

Dick, M. H., A. Herrera-Cubilla, and J. B. C. Jackson. 2003. Molecular phylogeny and phylogeography of free-living Bryozoa (Cupuladriidae) from both sides of the Isthmus of Panama. Molecular Phylogenetics and Evolution 27:355–371.

Dick, M. H., M. Hirose, R. Takashima, T. Ishimura, H. Nishi, and S. F. Mawatari. 2008. Application of MART analysis to infer paleoseasonality in a Pleistocene shallow marine benthic environment. Pages 93–99 *in* H. Okada, S. F. Mawatari, N. Suzuki, and P. Gautam, editors. Origin and evolution of natural diversity. Cambridge University Press.

Dick, M. H., T. Komatsu, R. Takashima, and A. N. Ostrovsky. 2014. A mid-Cretaceous (Albian–Cenomanian) shell-rubble bryozoan fauna from the Goshoura Group, Kyushu, Japan. Journal of Systematic Palaeontology 12:401–425.

Dick, M. H., S. Lidgard, D. P. Gordon, and S. F. Mawatari. 2009. The origin of ascophoran bryozoans was historically contingent but likely. Proceedings of the Royal Society of London, B 276:3141–3148.

Dick, M. H., C. Sakamoto, and T. Komatsu. 2018. Cheilostome Bryozoa from the Upper Cretaceous Himenoura Group, Kyushu, Japan. Paleontological Research 22:239–264.

Dominici, S. 2001. Taphonomy and paleoecology of shallow marine macrofossil assemblages in a collisional setting (Late Pliocene-Early Pleistocene, Western Emilia, Italy). Palaios 16:336–353.

Dudley, J. E. 1973. Observations on the reproduction, early larval development, and colony astogeny of *Conopeum tenuissimum* (Canu). Chesapeake Science 14:270–278.

Dumont, J. P. C. 1981. A report on the cheilostome Bryozoa of the Sudanese Red Sea. Journal of Natural History 15:623–637.

Duncan, H. 1939. Trepostomatous Bryozoa from the Traverse Group of Michigan. Contributions from the Museum of Paleontology, University of Michigan 5:171–270.

Dzik, J. 1975. The origin and early phylogeny of the cheilostomatous Bryozoa. Acta Palaeontologica Polonica 20:395–423.

Dzik, J. 1992. Early astogeny and relationships of the Ordovician rhabdomesine bryozoans. Acta Palaeontologica Polonica 37:37–54.

D'Hondt, J.-L. 2006. Nouvelle explications des planches de "Polypes" de la Description de l'Égypte. II - Bryozoaires. Collection Nouvelle Description de l'Egypte 2:1–86.

D'Onghia, G., F. Capezzuto, F. Cardone, R. Carlucci, A. Carluccio, G. Chimienti, G. Corriero, C. Longo, P. Maiorano, F. Mastrototaro, P. Panetta, A. Rosso, R. Sanfilippo, L. Sion, and A. Tursi. 2015. Macro- and megafauna recorded in the submarine Bari Canyon (southern Adriatic, Mediterranean Sea) using different tools. Mediterranean Marine Science 16:180–196.

Eliášová, H. 2005. Upper Cenomanian-Turonian two species of cyclostomaceous Bryozoa from the northern part of the Bohemian Cretaceous basin, Czech Republic. Journal of the Czech Geological Society 50:59–62.

Ernst, A. 2000. Permian Bryozoans of the NW-Tethys. Facies 43:79–102.

Ernst, A. 2009. *Petaloporella* (Cryptostoma, Bryozoa) from the Lower Devonian of central Bohemia. Bulletin of Geosciences 84:767–770.

Ernst, A. 2013. Diversity dynamics and evolutionary patterns of Devonian Bryozoa. Palaeobiodiversity and Palaeoenvironments 93:45–63.

Ernst, A., and M. Carrera. 2008. Cryptostomid bryozoans from the Sassito Formation, Upper Ordovician cool-water carbonates of the Argentinean Precordillera. Palaeontology 51:1117–1127.

Ernst, A., and P. Königshof. 2008. The role of bryozoans in fossil reefs—an example from the Middle Devonian of the Western Sahara. Facies 54:613–620.

Ernst, A., and P. Königshof. 2010. Bryozoan fauna and microfacies from a Middle Devonian reef complex (Western Sahara, Morocco). Abhandlungen der Senckenberg Gesellschaft Naturforschung 568:1–91.

Ernst, A., P. Königshof, P. D. Taylor, and J. Bohaty. 2011. Microhabitat complexity—an example from Middle Devonian bryozoan-rich sediments in the Blankenheim Syncline (northern Eifel, Rheinisches Schiefergebirge). Palaeobiodiversity and Palaeoenvironments 91:257–284.

Ernst, A., and A. May. 2012. Bryozoan fauna from the Lower Devonian (Middle Lochkovian) of Sierra de Guadarrama, Spain. Journal of Paleontology 86:60–80.

Ernst, A., A. May, and S. Marks. 2012. Bryozoans, corals, and microfacies of Lower Eifelian (Middle Devonian) limestones at Kierspe, Germany. Facies 58:727–758.

Ernst, A., and E. Minwegen. 2006. Late Carboniferous bryozoans from La Hermida, Spain. Acta Palaeontologica Polonica 51:569–588.

Ernst, A., A. Munnecke, and I. Oswald. 2015a. Exceptional bryozoan assemblage of a microbial-dominated reef from the early Wenlock of Gotland, Sweden. GFF 0:1–24.

Ernst, A., P. Schäfer, and J. A. Grant-Mackie. 2015b. New Caledonian Triassic Bryozoa. Journal of Paleontology 89:730–747.

Ernst, A., P. Schäfer, and J. J. G. Reijmer. 2005. Stenolaemate Bryozoa from the Upper Carboniferous of the Cantabrian Basin, northern Spain. Senckenbergiana lethaea 85:301–317.

Ernst, A., B. Senowbari-Daryan, and K. Rashidi. 2009. Bryozoa from the Surmaq Formation (Permian) of the Hambast Mountains, south of Abadeh, central Iran. Facies 55:595–608.

Ernst, A., O. Weidlich, and P. Schäfer. 2008. Stenolaemate Bryozoa from the Permian of Oman (Aseelah Unit, Batain coast). Journal of Paleontology 82:676–716.

Ernst, A., and C. F. Winkler Prins. 2008. Pennsylvanian bryozoans from the Cantabrian Mountains (northwestern Spain). Scripta Geologica 137:1–123.

Ferrario, J., J.-L. D'Hondt, A. Marchini, and A. Occhipinti-Ambrogi. 2015. From the Pacific Ocean to the Mediterranean Sea: *Watersipora arcuata*, a new non-indigenous bryozoan in Europe. Marine Biology Research 11:909–919.

Fordinál, K., K. Zágorsek, and A. Zlinská. 2006. Early Sarmatian biota in the northern part of the Danube Basin (Slovakia). Geologica Carpathica 57:123–130.

Frazier, J. G., J. E. Winston, and C. A. Ruckdeschel. 1992. Epizoan communities on marine turtles. III. Bryozoa. Bulletin of Marine Science 51:1–8.

Fritz, M. A. 1944. Upper Devonian Bryozoa from New Mexico. Journal of Paleontology 18:31–41.

Fritz, M. A. 1946. Permian(?) Bryozoa from Sustut Lake, British Columbia. Journal of Paleontology 20:86–87.

Galle, A., and R. L. Parsley. 2005. Epibiont relationships on hyolithids demonstrated by Ordovician trepostomes (Bryozoa) and Devonian tabulates (Anthozoa). Bulletin of Geosciences 80:125–138.

Gautier, T. G., P. N. Wyse Jackson, and F. K. McKinney. 2013. *Adlatipora*, a distinctive new Acanthocladiid Bryozoan from the Permian of the Glass Mountains, Texas, U.S.A., and its bearing on fenestrate astogeny and growth. Journal of Paleontology 87:444–455.

Gazdzicki, A., A. Tatur, U. Hara, and R. A. del Valle. 2004. The Weddell Sea Formation: post-Late Pliocene terrestrial glacial deposits on Seymour Island, Antarctic Peninsula. Polish Polar Research 25:189–204.

Gili, J.-M., R. Coma, C. Orejas, P. J. López-González, and M. Zabala. 2001. Are Antarctic suspension-feeding communities different from those elsewhere in the world? Polar Biology 24:473–485.

Gilmour, E. H. 2007. New Carboniferous Bryozoa of the Bird Spring Formation, southern Nevada. Journal of Paleontology 81:581–587.

Gilmour, E. H., and M. E. McColloch. 1995. Fenestrida and Rhabdomesida (Bryozoa) of the Otter Formation (Viséan), Central Montana. Journal of Paleontology 69:813–830.

Gilmour, E. H., M. E. McColloch, and B. R. Wardlaw. 1997. Bryozoa of the Murdock Mountain Formation (Wordian, Permian), Leach Mountains, Northeastern Nevada. Journal of Paleontology 71:214–236.

Gilmour, E. H., and E. M. Snyder. 2000. Bryozoa of the Mission Argillite (Permian) Northeastern Washington. Journal of Paleontology 74:545–570.

Gontar, V. I. 2009. New species from the Order Cyclostomata (Bryozoa) from the northern and middle Kurile Islands. Proceedings of the Zoological Institute RAS 313:41–57.

Gordon, D. P. 1988. The bryozoan families Sclerodomidae, Bifaxariidae, and Urceoliporidae and a novel type of frontal wall. New Zealand Journal of Zoology 15:249–290.

Gordon, D. P. 1989. The marine fauna of New Zealand: Bryozoa: Gymnolaemata (Cheilostomatida Ascophorina) from the western South Island continental shelf and slope. New Zealand Oceanographic Institute Memoir 97:1–158.

Gordon, D. P. 1993a. Bryozoa: The ascophorine infraorders Cribriomorpha, Hippothoomorpha and Umbonulomorpha mainly from New Caledonian waters. Mémoires du Muséum National d'Histoire Naturelle 158:299–347.

Gordon, D. P. 1993b. Bryozoan frontal shields: studies on umbonulomorphs and impacts on classification. Zoologica Scripta 22:203–221.

Gordon, D. P. 2000. First fossil occurrence of the austral bryozoan family Urceoliporidae. New Zealand Journal of Geology and Geophysics 43:385–389.

Gordon, D. P. 2009a. New names for some bryozoan homonyms. Zootaxa 2133:64–68.

Gordon, D. P. 2009b. *Baudina* gen. nov., constituting the first record of Pasytheidae from Australia, and Sinoflustridae fam. nov., with a checklist of Bryozoa and Pterobranchia from Beagle Gulf. The Beagle, Records of the Museums and Art Galleries of the Northern Territory 25:43–54.

Gordon, D. P. 2009c. New bryozoan taxa from a new marine conservation area in New Zealand, with a checklist of Bryozoa from Greater Cook Strait. Zootaxa 1987:39–60.

Gordon, D. P. 2014. Apprehending novel biodiversity—fifteen new genera of Zealandian Bryozoa. Journal of the Marine Biological Association of the United Kingdom 94:1597–1628.

Gordon, D. P., and A. V. Grischenko. 1994. Bryozoan frontal shields: the types species of *Desmacystis, Ramphostomella, Rhamphosmittina, Rhamphostomellina*, and new genus *Arctonula*. Zoologia Scripta 23:61–72.

Gordon, D. P., S. F. Mawatari, and H. Kajihara. 2002. New taxa of Japanese and New Zealand Eurystomellidae (Phylum Bryozoa) and their phylogenetic relationships. Zoological Journal of the Linnean Society 136:199–216.

Gordon, D. P., and W. B. Rudman. 2006. *Integripelta acanthus* n. sp. (Bryozoa: Eurystomellidae) — a tropical prey species of *Okenia hiroi* (Nudibranchia). Zootaxa 1229:41–48.

Gordon, D. P., and M. E. Spencer Jones. 2013. The amathiiform Ctenostomata (phylum Bryozoa) of New Zealand - including four new species, two of them of probable alien origin. Zootaxa 3647:75–95.

Gordon, D. P., I. G. Stuart, and J. D. Collen. 1994. Bryozoan fauna of the Kaipuke Siltstone, northwest Nelson: a Miocene homologue of the modern Tasman Bay coralline bryozoan grounds. New Zealand Journal of Geology and Geophysics 37:239–247.

Gordon, D. P., and P. D. Taylor. 2008. Systematics of the bryozoan genus *Macropora* (Cheilostomata). Zoological Journal of the Linnean Society 153:115–146.

Gordon, D. P., and P. D. Taylor. 2010. New seamount- and ridge-associated cyclostome Bryozoa from New Zealand. Zootaxa 2533:43–68.

Gordon, D. P., and P. D. Taylor. 2015. Bryozoa of the Early Eocene Tumaio Limestone, Chatham Island, New Zealand. Journal of Systematic Palaeontology 13:983–1070.

Gordon, D. P., P. D. Taylor, and F. P. Bigey. 2009. Phylum Bryozoa - moss animals, sea mats, lace corals. Pages

271–297 *in* D. P. Gordon, editor. The New Zealand inventory of biodiversity. Volume 1. Kingdom Animalia. Radiata, Lophotrochozoa, and Deuterostomia. Canterbury University Press, Christchurch.

Gorjunova, R. V., and A. V. Koromyslova. 2008. A new genus of the Arenigian bryozoans, Lynnopora, and its systematic position in the family Revalotrypidae Gorjunova, 1988. Paleontological Journal 42:491–499.

Gómez, A., P. J. Wright, D. H. Lunt, J. M. Cancino, G. R. Carvalho, and R. N. Hughes. 2007. Mating trials validate the use of DNA barcoding to reveal cryptic speciation of a marine bryozoan taxon. Proceedings of the Royal Society of London, B 274:199–207.

Grange, K. R., and D. P. Gordon. 2005. The spread and potential impact of the introduced bryozoan *Biflustra grandicella*. National Institute of Water and Atmospheric Research Final Research Report 31.

Greeley, R. 1969. Basally "uncalcified" zoaria of lunulitiform Bryozoa. Journal of Paleontology 43:252–256.

Gregory, J. W. 1896. Catalogue of the fossil Bryozoa in the Department of Geology, British Museum (Natural History). The Jurassic Bryozoa. Pages 1–239. Trustees of the British Museum, London.

Grischenko, A. V., M. A. Dick, and S. F. Mawatari. 2007. Diversity and taxonomy of intertidal Bryozoa (Cheilostomata) at Akkeshi Bay, Hokkaido, Japan. Journal of Natural History 41:1047–1161.

Grischenko, A. V., D. P. Gordon, A. Nojo, M. Kawamura, N. Kaneko, and S. F. Mawatari. 2004. New cheilostome bryozoans from the Middle Miocene of Hokkaido, Japan, and the first fossil occurrences of *Kubaninella* and *Hayamiellina* gen. nov. Paleontological Research 8:167–179.

Grischenko, A. V., S. F. Mawatari, and P. D. Taylor. 2000. Systematics and phylogeny of the cheilostome bryozoan *Doryporella*. Zoologia Scripta 29:247–264.

Grischenko, A. V., J. E. Seo, and B. S. Min. 2010. *Flustrellidra armata* (Bryozoa: Ctenostomatida) — a new species from the southern shoreline of Korea. Zootaxa 2684:25–35.

Guha, A. K. 2013. Tertiary Bryozoa from western Kachchh, Gujarat - a review. Journal of the Palaeontological Society of India 58:3–15.

Guha, A. K., and K. Gopikrishna. 2005. New smittinid (Bryozoa, Cheilostomata) species from Tertiary sequences of western Kachchh, Gujarat, India. Freiberger Forschungshefte C507:11–25.

Guha, A. K., and K. Gopikrishna. 2007a. Some fossil ascophoran bryozoan taxa from Tertiary sequences of Western Kachchh, Gujarat. Journal of the Paleontological Society of India 52:195–222.

Guha, A. K., and K. Gopikrishna. 2007b. New calloporid (Bryozoa, Cheilostomata) species from Tertiary sequences of western Kachchh, Gujarat. Journal of the Geological Society of India 70:121–130.

Guha, A. K., and K. Gopikrishna. 2007c. New celleporid (Bryozoa, Cheilostomata) species from Tertiary deposits of western Kachchh, Gujarat, India. Paläontologische Zeitschrift 81:83–92.

Hageman, S. J. 1991a. *Worthenopora*: an unusual cryptostome (Bryozoa) that looks like a cheilostome. Journal of Paleontology 65:648–661.

Hageman, S. J. 1991b. Approaches to systematic and evolutionary studies of perplexing groups: an example using fenestrate Bryozoa. Journal of Paleontology 65:630–647.

Hageman, S. J. 1993. Effects of nonnormality on studies of morphological variation of a rhabdomesine bryozoan, *Streblotrypa* (*Streblascopora*) *prisca* (Gabb and Horn). The University of Kansas Paleontological Contributions 4:1–13.

Hageman, S. J., P. E. Bock, Y. Bone, and B. Mcgowran. 1998. Bryozoan growth habits: Classification and analysis. Journal of Paleontology 72:418–436.

Hageman, S. J., J. Lukasik, B. McGowran, and Y. Bone. 2003. Paleoenvironmental significance of Celleporaria (Bryozoa) from modern and Tertiary cool-water carbonates of southern Australia. Palaios 18:510–527.

Hageman, S. J., and F. K. McKinney. 2010. Discrimination of fenestrate bryozoan genera in morphospace. Palaeontologia Electronica 13:1–43.

Hageman, S. J., P. N. Wyse Jackson, A. R. Abernethy, and M. Steinthorsdottir. 2011. Calender scale, environmental variation preserved in the skeletal phenotype of a fossil bryozoan (*Rhombopora blakei* n.sp.), from the Mississippian of Ireland. Journal of Paleontology 85:853–870.

Hao, J., C. Li, X. Sun, and Q. Yang. 2005. Phylogeny and divergence time estimation of cheilostome bryozoans based on mitochodrial 16S rRNA sequences. Chinese Science Bulletin 50:1205–1211.

Hara, U. 2001. Bryozoans from the Eocene of Seymour Island, Antarctic Peninsula. Palaeontological Results of the Polish Antarctic Expeditions. Part III. Palaeontologia Polonica 60:33–156.

Hara, U. 2002. A new macroporid bryozoan from Eocene of Seymour Island, Antarctic Peninsula. Polish Polar Research 23:213–225.

Hara, U., A. Ernst, and Z. Mikolajewski. 2009. Permian trepostome bryozoans from the Zechstein Main Dolomite (Ca2) of Western Poland and NE Germany. Geological Quartelry 53:249–254.

Hara, U., and M. Jasionowski. 2012. The Early Sarmatian bryozoan *Celleporina medoborensis* sp. nov. from the Medobory reefs of western Ukraine (Central Paratethys). Geological Quarterly 56:895–906.

Harmelin, J.-G. 1988. Les Bryozoaires, de bons indicateurs bathymétriques en paléoécologie? Géologie Méditerranéenne 15:49–63.

Harmelin, J.-G. 2014. *Monoporella bouchardii* (Audouin & Savigny, 1826) (Bryozoa, Cheilostomata): a forgotten taxon redescribed from Eastern Mediterranean material. Cahiers de Biologie Marine 55:91–99.

Harmelin, J.-G., G. Bitar, and H. Zibrowius. 2009. Smittinidae (Bryozoa, Cheilostomata) from coastal habitats of Lebanon (Mediterranean Sea), including new and non-indigenous species. Zoosystema 31:163–187.

Harmelin, J.-G., A. N. Ostrovsky, J. P. Cáceres-Chamizo, and J. Sanner. 2011. Bryodiversity in the tropics: taxonomy of *Microporella* species (Bryozoa, Cheilostomata) with personate maternal zooids from Indian Ocean, Red Sea and southeast Mediterranean. Zootaxa 2798:1–30.

Harmer, S. F. 1900. A revision of the genus *Steganoporella*. Quarterly Journal of Microscopical Science 43:225–297.

Harmer, S. F. 1902. On the morphology of the Cheilostomata. Quarterly Journal of Microscopical Science 46:263–350.

Harmer, S. F. 1915. The Polyzoa of the Siboga Expedition, Part I. Entoprocta, Ctenostomata and Cyclostomata. Siboga Expeditie 28a:1–180.

Harzhauser, M., A. Kroh, O. Mandic, W. E. Piller, U. Göhlich, M. Reuter, and B. Berning. 2007. Biogeographic responses to geodynamics: a key study all around the Oligo–Miocene Tethyan Seaway. Zoologischer Anzeiger 246:241–256.

Harzhauser, M., and W. E. Piller. 2004. The Early Sarmatian - hidden seesaw changes. Cour. Forsch.-Inst. Senckenberg 246:89–111.

Hastings, A. B. 1949. On the Polyzoan *Cellepora pulchra* Manzoni and the genus *Hippopleurifera*. Journal of the Linnean Society of London, Zoology 41:521–528.

Hastings, A. B. 1964. The cheilostomatous Polyzoa *Neoeuthyris woosteri* (MacGillivray) and *Reginella doliaris* (Maplestone). Bulletin of the British Museum (Natural History), Zoology 11:245–262.

Hayward, P. J. 1974. Studies on the cheilostome bryozoan fauna of the Aegean island of Chios. Journal of Natural History 8:369–402.

Hayward, P. J. 1978. Bryozoa from the west European continental slope. Journal of Zoology, London 184:207–224.

Hayward, P. J. 1993. New species of cheilostomate Bryozoa from Antarctica and the Subantarctic southwest Atlantic. Journal of Natural History 27:1409–1430.

Hayward, P. J., and K. B. Hansen. 1999. Three newly recognized cheilostomate bryozoans from the British sea area. Journal of the Marine Biological Association 79:917–921.

Hayward, P. J., and J. S. Ryland. 1985. Systematic note on some British Cyclostomata (Bryozoa). Journal of Natural History 19:1073–1078.

Hayward, P. J., and J. S. Ryland. 1990. Some Antarctic and Subantarctic species of Microporellidae (Bryozoa: Cheilostomata). Journal of Natural History 24:1263–1287.

Hayward, P. J., and P. D. Taylor. 1984. Fossil and Recent Cheilostomata (Bryozoa) from the Ross Sea, Antarctica. Journal of Natural History 18:71–94.

Hayward, P. J., and J. P. Thorpe. 1988a. Species of *Chaperiopsis* (Bryozoa, Cheilostomata) collected by Discovery

Investigations. Journal of Natural History 22:45–69.

Hayward, P. J., and J. P. Thorpe. 1988b. Species of *Arachnopusia* (Bryozoa: Cheilostomata) collected by Discovery Investigations. Journal of Natural History 22:773–799.

Hayward, P. J., and J. E. Winston. 2011. Bryozoa collected by the United States Antarctic Research Program: new taxa and new records. Journal of Natural History 45:2259–2338.

Herrera-Cubilla, A., M. H. Dick, J. Sanner, and J. B. C. Jackson. 2006. Neogene Cupuladriidae of tropical America. I: Taxonomy of Recent Cupuladria from opposite sides of the Isthmus of Panama. Journal of Paleontology 80:245–263.

Herrera-Cubilla, A., M. H. Dick, J. Sanner, and J. B. C. Jackson. 2008. Neogene Cupuladriidae of tropical America. II: Taxonomy of Recent Discoporella from opposite sides of the Isthmus of Panama. Journal of Paleontology 82:279–298.

Herrera-Cubilla, A., and J. B. Jackson. 2014. Phylogeny of genus *Cupuladria* (Bryozoa, Cheilostomata) in the Neogene of tropical America. Journal of Paleontology 88:851–894.

Hillmer, G. 2006. Erhard Voigt, 28. Juli 1905 - 22. November 2004. Paläontologische Zeitschrift 80:1–11.

Hinds, R. W. 1975. Growth mode and homeomorphism in cyclostome Bryozoa. Journal of Paleontology 49:875–910.

Holcová, K., and K. Zágorsek. 2008. Bryozoa, foraminifera and calcareous nannoplankton as environmental proxies of the “bryozoan event” in the Middle Miocene of the Central Paratethys (Czech Republic). Palaeogeography, Palaeoclimatology, Palaeoecology 267:216–234.

Holcová, K., K. Zágorsek, V. Jasková, and T. Lehotsky. 2007. The oldest Miocene Bryozoa from the Carpathian Foredeep (boreholes Premyslovice). Scripta Facultatis Scientiarum Naturalium Universitatis Masarykianae Brunensis 36:47–55.

Horowitz, A. S., and J. F. Pachut. 1993. Specific, generic, and familial diversity of Devonian bryozoans. Journal of Paleontology 67:42–52.

Hunter, E., K. Shimizu, and N. Fusetani. 1999. Role of protein in larval swimming and metamorphosis of *Bugula neritina* (Bryozoa: Cheilostomatida). Marine Biology 133:701–707.

Hurlbut, C. J. 1991. Community recruitment: settlement and juvenile survival of seven co-occurring species of sessile marine invertebrates. Marine Biology 109:507–515.

Illies, G. 1981. Evolutionary trends in budding patterns of stomatoporid cyclostomatous Bryozoa and *Stomatoporopsis* n. gen. Oberrheinische geologische Abhandlungen 30:27–42.

Illies, G. 1987. A new uniserial *Stomatopora* (Bryozoa Cyclostomata) from the Upper Cretaceous of northern Germany. Pages 129–136 *in* J. R. P. Ross, editor. Bryozoa: Present and past. Western Washington University, Bellingham.

Jablonski, D., S. Lidgard, and P. D. Taylor. 1997. Comparative ecology of bryozoan radiations: origin of novelties in cyclostomes and cheilostomes. Palaios 12:505–523.

Jackson, J. B. C., and A. O'Dea. 2013. Timing of the oceanographic and biological isolation of the Caribbean Sea from the tropical eastern Pacific Ocean. Bulletin of Marine Science 89:779–800.

Jackson, J. B. C., J. E. Winston, and A. G. Coates. 1985. Niche breadth, geographic range, and extinction of caribbean reef-associated cheilostome Bryozoa and Scleractinia. Proceedings of the Fifth International Coral Reef Congress 4:151–158.

Jackson, P. N. W., M. M. Key, and S. P. Coakley. 2014. Epizoozoan trepostome bryozoans on nautiloids from the Upper Ordovician (Katian) of the Cincinnati Arch region, USA: An assessment of growth, form, and water flow dynamics. Journal of Paleontology 88:475–487.

Jagadeeshan, S., and A. O'Dea. 2012. Integrating fossils and molecules to study cupuladriid evolution in an emerging isthmus. Evolutionary Ecology 26:337–355.

James, D. W., M. S. Foster, and J. O'Sullivan. 2006. Bryoliths (Bryozoa) in the Gulf of California. Pacific Science 60:117–124.

James, N. P., Y. Bone, L. B. Collins, and T. K. Kyser. 2001. Surficial sediments of the Great Australian Bight:

facies dynamics and oceanography on a vast cool-water carbonate shelf. Journal of Sedimentary Research 71:549–567.

James, N. P., D. A. Feary, C. Betzler, Y. Bone, A. E. Holbourn, Q. Li, H. Machiyama, J. A. T. Simo, and F. Surlyk. 2004. Origin of late Pleistocene bryozoan reef mounds: Great Australian Bight. Journal of Sedimentary Research 74:20–48.

Jelly, E. C. 1889. A synonymic catalogue of the Recent marine Bryozoa. Including fossil synonyms. Dulau & Company, London.

Jiménez-Sánchez, A., P. D. Taylor, and J. B. Gómez. 2013. Palaeogeographical patterns in Late Ordovician bryozoan morphology as proxies for temperature. Bulletin of Geosciences 88:417–426.

Johnson, M. E., R. S. Ramalho, B. G. Baarli, M. Cachão, C. M. da Silva, E. Mayoral, and A. Santos. 2014. Miocene–Pliocene rocky shores on São Nicolau (Cape Verde Islands): contrasting windward and leeward biofacies on a volcanically active oceanic island. Palaeogeography, Palaeoclimatology, Palaeoecology 395:131–143.

Jürgensen, T. 1968. Cheilostome Bryozoa from the Danian deposits at Vallensbaek, Denmark. Meddelelser fra Dansk Geologisk Forening 18:187–204.

Jürgensen, T. 1971. *Tornipora*, a new cheilostome bryozoan from the Maastrichtian and Danian of Denmark. Bulletin of the Geological Society of Denmark 20:390–396.

Kahle, J., G. Liebezeit, and G. Gerdes. 2003. Growth aspects of *Flustra foliacea* (Bryozoa, Cheilostomata) in laboratory culture. Hydrobiologia 503:237–244.

Kaiser, S., H. J. Griffiths, D. K. A. Barnes, S. N. Brandão, A. Brandt, and P. E. O'Brien. 2011. Is there a distinct continental slope fauna in the Antarctic? Deep-Sea Research Part II: Topical Studies in Oceanography 58:91–104.

Karklins, O. L. 1983. Ptilodictyoid Cryptostomata Bryozoa from the Middle and Upper Ordovician rocks of central Kentucky. The Paleontological Society Memoir 14:1–31.

Keij, A. J. 1972. *Sylonika* and *Kylonisa*, two new Palaeogene bryozoan genera (Cheilostomata, Skyloniidae). Scripta Geologica 11:1–15.

Key, M. M. 2002. Presentation of the Paleontological Society Medal to Alan H. Cheetham. Journal of Paleontology 76:783.

Key, M. M., P. N. Wyse Jackson, and S. H. Felton. 2016. Intracolony variation in colony morphology in reassembled fossil ramose stenolaemate bryozoans from the Upper Ordovician (Katian) of the Cincinnati Arch region, USA. Journal of Paleontology 90:400–412.

Key, M. M., P. N. Wyse Jackson, E. Håkansson, W. P. Patterson, and M. D. Moore. 2005. Gigantism in Permian trepostomes from Greenland: testing the algal symbiosis hypothesis using δ13C and δ18O values. Pages 141–151 *in* H. I. Moyano, J. M. Cancino, and P. N. Wyse Jackson, editors. Bryozoan studies 2005. Balkema, Leiden.

Key, M. M., P. N. Wyse Jackson, and L. J. Vitiello. 2011. Stream channel network analysis applied to colony-wide feeding structures in a Permian bryozoan from Greenland. Paleobiology 37:287–302.

Kidwell, S. M., and E. D. Gyllenhaal. 1998. Symbiosis, competition, and physical disturbance in the growth histories of Pliocene cheilostome bryoliths. Lethaia 31:221–239.

Kiepura, M. 1962. Bryozoa from the Ordovician erratic boulders of Poland. Acta Palaeontologica Polonica 7:347–428.

Knowles, T., M. J. Leng, M. Williams, P. D. Taylor, H. J. Sloane, and B. Okamura. 2010. Interpreting seawater temperature range using oxygen isotopes and zooid size variation in Pentapora foliacea (Bryozoa). Marine Biology 157:1171–1180.

Koçak, F. 2007. A new alien bryozoan *Celleporaria brunnea* (Hincks, 1884) in the Aegean Sea (eastern Mediterranean). Scientia Marina 71:191–195.

Koçak, F., and A. Önen. 2014. Checklist of Bryozoa on the coasts of Turkey. Turkish Journal of Zoology 38:880–891.

Koenig, J. W. 1958. Fenestrate Bryozoa in the Chouteau Group of central Missouri. Journal of Paleontology 32:126–143.

Koromyslova, A. V. 2014a. Morphological features and systematic position of the bryozoans *Onychocella rowei* and *O. Mimosa* (Cheilostomata) from a Campanian erratic block (Belarus). Paleontological Journal 48:275–286.

Koromyslova, A. V. 2014b. The earliest calcified opercula of bryozoans of the order Cheilostomata. Paleontological Journal 48:587–593.

Koromyslova, A. V., and A. V. Pakhnevich. 2016. New species of *Pachydermopora* Gordon, 2002 and *Beisselina* Canu, 1913 (Bryozoa: Cheilostomida) from a Campanian erratic block (Belarus) and their micro-CT investigation. Paleontological Journal 50:41–53.

Koromyslova, A. V., and E. A. Shcherbinina. 2015. New data on the morphology and age of the bryozoans of the genus *Onychocella* from the Campanian-Maastrichtian of Turkmenistan and Uzbekistan. Paleontological Journal 49:474–484.

Kuklinski, P. 2013. Biodiversity and abundance patterns of rock encrusting fauna in a temperate fjord. Marine Environmental Research 87-88:61–72.

Kuklinski, P., A. V. Grischenko, and S. C. Jewett. 2015. Two new species of the cheilostome bryozoan *Cheilopora* from the Aleutian Islands. Zootaxa 3963:434–442.

Kuklinski, P., B. Gulliksen, O. J. Lønne, and J. M. Weslawski. 2006. Substratum as a structuring influence on assemblages of Arctic bryozoans. Polar Biology 29:652–661.

Lagaaij, R. 1959. Some species of Bryozoa new to the Bowden Beds, Jamaica, B.W.I. Micropaleontology 5:482–486.

Landing, E., J. B. Antcliffe, M. D. Brasier, and A. B. English. 2015. Distinguishing Earth's oldest known bryozoan (Pywackia, late Cambrian) from pennatulacean octocorals (MesozoicRecent). Journal of Paleontology 89:292–317.

Landing, E., A. English, and J. D. Keppie. 2010. Cambrian origin of all skeletalized metazoan phyla - discovery of Earth's oldest bryozoans (Upper Cambrian, southern Mexico). Geology 38:547–550.

Larwood, G. P. 1962. The morphology and systematics of some Cretaceous cribrimorph Polyzoa (Pelmatoporinae). Bulletin of the British Museum (Natural History), Geology 6:1–285.

Larwood, G. P. 1969. Frontal calcification and its function on some Cretaceous and Recent cribrimorph and other cheilostome Bryozoa. Bulletin of the British Museum (Natural History), Zoology 18:171–182.

Larwood, G. P., A. W. Medd, D. E. Owen, and R. Tavener-Smith. 1967. Bryozoa. Pages 295–379 *in* The Fossil Record. Geological Society of London, London.

Lidgard, S., M. C. Carter, M. H. Dick, D. P. Gordon, and A. N. Ostrovsky. 2011. Division of labor and recurrent evolution of polymorphisms in a group of colonial animals. Evolutionary Ecology 26:233–257.

Lidgard, S., and J. B. Jackson. 1989. Growth in encrusting cheilostome bryozoans: I. Evolutionary trends. Paleobiology 15:255–282.

Livingstone, A. A. 1926. Studies on Australian Bryozoa. No. 4. Records of the Australian Museum 15:167–176.

Lodola, A., J. Ferrario, and A. Occhipinti-Ambrogi. 2015. Further Mediterranean expansion of the non-indigenous bryozoan *Celleporaria brunnea*: multiple records along the Italian coasts. Scientia Marina 79:263–274.

Lombardi, C., S. Cocito, K. Hiscock, A. Occhipinti Ambrogi, M. Setti, and P. D. Taylor. 2008. Influence of seawater temperature on growth bands, mineralogy and carbonate production in a bioconstructional bryozoan. Facies 54:333–342.

Lombardi, C., S. Cocito, A. Occhipinti Ambrogi, and K. Hiscock. 2006. The influence of seawater temperature on zooid size and growth rate in *Pentapora fascialis* (Bryozoa: Cheilostomata). Marine Biology 149:1103–1109.

Lombardi, C., M. C. Gambi, C. Vasapollo, P. D. Taylor, and S. Cocito. 2011. Skeletal alterations and polymorphism in a Mediterranean bryozoan at natural CO_2 vents. Zoomorphology 130:135–145.

Lombardi, C., P. D. Taylor, and S. Cocito. 2010. Systematics of the Miocene–Recent bryozoan genus *Pentapora* (Cheilostomata). Zoological Journal of the Linnean Society 160:17–39.

Lonsdale, W. 1845a. Corals from the Tertiary formations of North America. I. Account of ten species of Polyparia obtained from the Miocene Tertiary formations of North America. Quarterly Journal of the Geological Society of London 1:495–509.

Lonsdale, W. 1845b. Account of six species of Polyparia obtained from Timber Creek, New Jersey. Quarterly Journal of the Geological Society of London 1:65–75.

López de la Cuadra, C. M., and J. C. García-Gómez. 2000. The cheilostomate Bryozoa (Bryozoa: Cheilostomatida) collected by the Spanish 'Antártida 8611' Expedition to the Scotia Arc and South Shetland Islands. Journal of Natural History 34:755–772.

López Gappa, J. J. 1989. Overgrowth competition in an assemblage of encrusting bryozoans settled on artificial substrata. Marine Ecology Progress Series 51:121–130.

Ma, J., P. D. Taylor, F.-S. Xia, and R. Zhan. 2015. The oldest known bryozoan: Prophyllodictya (Cryptostomata) from the lower Tremadocian (Lower Ordovician) of Liujiachang, south-western Hubei, central China. Palaeontology 58:925–934.

Machiyama, H., T. Yamada, N. Kaneko, Y. Iryo, K. Odawara, R. Asami, H. Matsuda, S. F. Mawatari, Y. Bone, and N. P. James. 2003. Carbon and oxygen isotopes of cool-water bryozoans from the Great Australian Bight and their paleoenvironmental significance. Pages 1–29 *in* A. C. Hine, D. A. Feary, and M. J. Malone, editors. Proceedings of the ocean drilling programme, scientific results.

Mackie, J. A. 2003. A molecular analysis of bryozoan dispersal. PhD thesis, University of Melbourne, Melbourne.

Mackie, J. A., J. A. Darling, and J. B. Geller. 2012. Ecology of cryptic invasions: latitudinal segregation among *Watersipora* (Bryozoa) species. Scientific Reports 2:1–10.

Malecki, J. 1964. On two new genera of Bryozoa Cheilostomata from the Tortonian of Poland. Acta Palaeontologica Polonica 9:499–512.

Marintsch, E. J. 1981. Taxonomic reevaluation of *Prasopora simulatrix* Ulrich (Bryozoa: Trepostomata). Journal of Paleontology 55:957–961.

Maturo, F. J. S., and T. J. M. Schopf. 1968. Ectoproct and entoproct type material: reexamination of species from New England and Bermuda named by A.E. Verrill, J.W. Dawson and E. Desor. Postilla 120:1–95.

McColloch, M. E., E. H. Gilmour, and E. M. Snyder. 1994. The order Fenestrata (Bryozoa) of the Toroweap Formation (Permian), southern Nevada. Journal of Paleontology 68:746–762.

McGhee, G. R., and F. K. McKinney. 2002. A theoretical morphologic analysis of ecomorphologic variation in *Archimedes* helical colony form. Palaios 17:556–570.

McKinney, F. K. 1983. Ectoprocta (Bryozoa) from the Permian Kaibab Formation, Grand Canyon National Park, Arizona. Fieldiana Geology, N.S. 13:1–17.

McKinney, F. K. 2009. Bryozoan-hydroid symbiosis and a new ichnogenus, *Caupokeras.* Ichnos 16:193–201.

McKinney, F. K., and D. W. Burdick. 2001. A rare, larval-founded colony of the bryozoan Archimedes from the Carboniferous of Alabama. Palaeontology 44:855–859.

McKinney, F. K., and A. Jaklin. 2001. Sediment accumulation in a shallow-water meadow carpeted by a small erect bryozoan. Sedimentary Geology 145:397–410.

McKinney, F. K., and J. Kríz. 1986. Lower Devonian Fenestrata (Bryozoa) of the Prague Basin, Barrandian Area, Bohemia, Czechoslovakia. Fieldiana 1368.

McKinney, F. K., M. R. A. Listokin, and C. D. Phifer. 1986. Flow and polypide distribution in the cheilostome bryozoan *Bugula* and their inference in *Archimedes.* Lethaia 19:81–93.

McKinney, F. K., and G. R. McGhee. 2003. Evolution of erect helical colony form in the Bryozoa: phylogenetic, functional, and ecological factors. Biological Journal of the Linnean Society 80:235–260.

McKinney, F. K., and M. J. McKinney. 1994. Preferences for settlement conditions by larvae of *Schizotheca serratimargo* (Hincks, 1886), an erect bryozoan from protected habitats. Pages 119–124 *in* P. J. Hayward, J. S. Ryland, and P. D. Taylor, editors. Biology and Palaeobiology of Bryozoans. Olsen & Olsen, Fredensborg.

McKinney, F. K., M. J. McKinney, and M. R. A. Listokin. 1987. Erect bryozoans are more than baffling: enhanced sedimentation rate by a living unilaminate branched bryozoan and possible implications for fenestrate bryozoan mudmounds. Palaios 2:41–47.

McKinney, F. K., and P. D. Taylor. 2001. Bryozoan generic extinctions and originations during the last one

hundred million years. Palaeontologia Electronica 4:1–26.

McKinney, F. K., and P. D. Taylor. 2003. Palaeoecology of free-lying domal bryozoan colonies from the Upper Eocene of southeastern USA. Acta Palaeontologica Polonica 48:447–462.

McKinney, F. K., P. D. Taylor, and V. A. Zullo. 1993. Lyre-shaped hornerid bryozoan colonies: homeomorphy in colony form between Paleozoic Fenestrata and Cenozoic Cyclostomata. Journal of Paleontology 67:343–354.

McKinney, F. K., and P. N. Wyse Jackson. 2010. *Phylloporina* Ulrich in Foerste, 1887 (Bryozoa, Fenestrata, Phylloporinina): proposed designation of *Retepora trentoniensis* Nicholson, 1875 as the type species. Bulletin of Zoological Nomenclature 67:38–43.

McKinney, F. K., and P. N. Wyse Jackson. 2015. Part G, Revised, Volume 2, Chapter 8H: Names of indeterminate, unrecognizable, or excluded genera previously assigned to Fenestrata. Treatise Online 64:1–5.

McShea, D. W., and E. P. Venit. 2002. Testing for bias in the evolution of coloniality: a demonstration in cyclostome bryozoans. Paleobiology 28:308–327.

Menzies, R. J. 1963. Abyssal Bryozoa collected by expeditions of the Lamont Geological Observatory. 1. Bicellariellidae (Bugulidae of authors), Kinetoskias. American Museum Novitates 2130:1–8.

Mesentseva, O. P. 2016. Cystoporida (Bryozoa) from the Emsian Stage (Lower Devonian) of the Salair Ridge and Gorny and Rudny Altai. Paleontological Journal 50:376–387.

Messina, C., M. A. Rosso, F. Sciuto, I. Di Geronimo, W. Nemec, T. Di Dio, R. Di Geronimo, R. Maniscalco, and R. Sanfilippo. 2007. Anatomy of a transgressive systems tract revealed by integrated sedimentological and palaeoecological study: the Barcellona Pozzo di Gotto Basin, northeastern Sicily, Italy. Pages 367–400 *in* G. Nichols, E. A. Williams, and C. Paola, editors. Sedimentary Processes, Environments and Basins – A Tribute to Peter Friend. Blackwell Publishing, Malden, MA, USA.

Moissette, P. 1996. The cheilostomate bryozoan *Batopora rosula* (Reuss, 1848): a paleobathymetric indicator in the Mediterranean Neogene. Pages 193–198 *in* D. P. Gordon, A. M. Smith, and J. A. Grant-Mackie, editors. Bryozoans in space and time. NIWA, Wellington.

Moissette, P. 2000. Changes in bryozoan assemblages and bathymetric variations. Examples from the Messinian of northwest Algeria. Palaeogeography, Palaeoclimatology, Palaeoecology 155:305–326.

Moissette, P., J.-J. Cornée, and E. Koskeridou. 2010. Pleistocene rolling stones or large bryozoan nodules in a mixed siliciclastic-carbonate environment (Rhodes, Greece). Palaios 25:24–39.

Moissette, P., A. Dulai, G. Escarguel, M. Kázmér, P. Müller, and J.-P. Saint Martin. 2007. Mosaic of environments recorded by bryozoan faunas from the Middle Miocene of Hungary. Palaeogeography, Palaeoclimatology, Palaeoecology 252:530–556.

Moissette, P., A. Dulai, and P. Müller. 2006. Bryozoan faunas in the Middle Miocene of Hungary: biodiversity and biogeography. Palaeogeography, Palaeoclimatology, Palaeoecology 233:300–314.

Moissette, P., and S. Pouyet. 1987. Bryozoan faunas and the Messinian salinity crisis. Annales Instituti Geologici Publici Hungarici 70:447–453.

Moissette, P., and J.-P. Saint-Martin. 1995. Bryozoaires des milieux récifaux miocènes du sillon sud-rifain au Maroc. Lethaia 28:271–283.

Montoya-Cadavid, E., P. Flórez-Romero, and J. E. Winston. 2007. Checklist of the marine Bryozoa of the Colombian Caribbean. Biota Colombiana 8:159–184.

Morozova, I. P., O. B. Weis, and G. Eacki. 2002. Emergence and extinction of the Givetian to Frasnian bryozoan faunas in the Kostomloty facies zone, Holy Cross Mountains, Poland. Acta Palaeontologica Polonica 47:307–317.

Moyano, H. I. 1983. Southern Pacific Bryozoa: a general view with emphasis on Chilean species. Gayana Zoologia 46:1–45.

Moyano, H. I. 1999. Magellan Bryozoa: a review of the diversity and of the Subantarctic and Antarctic zoogeographical links. Scientia Marina 63:219–226.

Mukai, H., K. Terakado, and C. G. Reed. 1997. Bryozoa. Pages 45–206 *in* F. W. Harrison and R. M. Woollacott, editors. Microscopic Anatomy of Invertebrates. Wiley-Liss, New York.

Nakrem, H. A., and N. Spjeldnaes. 1995. *Ramipora hochstetteri* Toula, 1875 (Bryozoa, Cystoporata), from the Permian of Svalbard. Journal of Paleontology 69:831–838.

Newton, G. B. 1971. Rhabdomesid bryozoans of the Wreford megacyclothem (Wolfcampian, Permian) of Nebraska, Kansas, and Oklahoma. University of Kansas Paleontological Contributions 56:1–71.

Nikulina, E. A. 2006. *Electra korobokkura* sp. n., a new species of cheilostome bryozoan from the Pacific coast of Hokkaido, Japan. Invertebrate Zoology 3:23–31.

Nikulina, E. A. 2007. *Einhornia*, a new genus for electrids formerly classified as the *Electra crustulenta* species group (Bryozoa, Cheilostomata). Schriften des Naturwissenschaftlichen Vereins für Schleswig-Holstein 69:29–40.

Nikulina, E. A. 2008a. Taxonomy and ribosomal DNA-based phylogeny of the *Electra crustulenta* species group (Bryozoa: Cheilostomata) with revision of Borg's varieties and description of *Electra moskvikvendi* sp. nov. from the western Baltic Sea. Organisms, Diversity & Evolution 8:215–229.

Nikulina, E. A. 2008b. *Electra scuticifera* sp. nov.: redescription of *Electra pilosa* from New Zealand as a new species (Bryozoa, Cheilostomata). Schriften des Naturwissenschaftlichen Vereins für Schleswig-Holstein 70:91–98.

Nikulina, E. A. 2010. Three new genera of Electridae (Bryozoa): *Arbopercula, Osburnea*, and *Arbocuspis*. Schriften des Naturwissenschaftlichen Vereins für Schleswig-Holstein 72:25–28.

Nikulina, E. A., A. N. Ostrovsky, and M. Claereboudt. 2012. A new species of the genus *Electra* (Bryozoa, Cheilostomata) from southern Oman, Arabian Sea. Pages 203–216 *in* A. Ernst, P. Schäfer, and J. Scholz, editors. Bryozoan studies 2010. Springer, Berlin.

Nikulina, E. N., R. Hanel, and P. Schäfer. 2007. Cryptic speciation and paraphyly in the cosmopolitan bryozoan *Electra pilosa* - impact of the Tethys closing on species evolution. Molecular Phylogenetics and Evolution 45:765–776.

Nitecki, M. H., and F. K. McKinney. 1975. *Cucumulites* Gurley 1884: a bryozoan, not a sponge. Journal of Paleontology 49:561–563.

Novak, V., N. Santodomingo, A. Rösler, E. Di Martino, J. C. Braga, P. D. Taylor, K. G. Johnson, and W. Renema. 2013. Environmental reconstruction of a late Burdigalian (Miocene) patch reef in deltaic deposits (East Kalimantan, Indonesia). Palaeogeography, Palaeoclimatology, Palaeoecology 374:110–122.

Nye, O. B., and D. V. Lemone. 1978. Multilaminar growth in *Reptomulticava texana*, a new species of cyclostome Bryozoa. Journal of Paleontology 52:830–845.

Okamura, B., A. O'Dea, P. D. Taylor, and A. Taylor. 2013. Evidence of El Niño/La Niña–Southern Oscillation variability in the Neogene-Pleistocene of Panama revealed by a new bryozoan assemblage-based proxy. Bulletin of Marine Science 89:857–867.

Olempska, E. 2012. Exceptional soft-tissue preservation in boring ctenostome bryozoans and associated "fungal" borings from the Early Devonian of Podolia, Ukraine. Acta Palaeontologica Polonica 57:925–940.

Osburn, R. C. 1919. Bryozoa of the Crocker Land Expedition. Bulletin of the American Museum of Natural History 41:603–624.

Osburn, R. C. 1950. Bryozoa of the Pacific Coast of America. Part 1, Cheilostomata-Anasca. Allan Hancock Pacific Expeditions 14:1–269.

Osburn, R. C. 1952. Bryozoa of the Pacific Coast of America. Part 2, Cheilostomata Ascophora. Allan Hancock Pacific Expeditions 14:271–611.

Osburn, R. C. 1953. Bryozoa of the Pacific Coast of America. Part 3, Cyclostomata, Ctenostomata, Entoprocta, and Addenda. Report of the Allan Hancock Pacific Expeditions 14:613–841.

Ostrovsky, A. N. 1998. Variability of ooeciostome shape in Antarctic idmidroniform bryozoans (Bryozoa: Cyclostomatida). Zoologischer Anzeiger 237:97–106.

Ostrovsky, A. N. 2004. Brood chambers (ovicells) of cheilostome bryozoans (Bryozoa: Gymnolaemata): structure, research history, and modern problematics. Russian Journal of Marine Biology 30:43–55.

Ostrovsky, A. N. 2008. External versus internal and self- versus cross-fertilization in Bryozoa: transformation of the view and evolutionary considerations. Pages 103–115 *in* P. N. Wyse Jackson and M. E. Spencer Jones,

editors. Annals of Bryozoology: Aspects of the History of Research on Bryozoans. International Bryozoology Association, Dublin.

Ostrovsky, A. N. 2013. Evolution of Sexual Reproduction in Marine Invertebrates. Example of Gymnolaemate Bryozoans. Page 356. Springer, Dordrecht.

Ostrovsky, A. N., D. P. Gordon, and S. Lidgard. 2009. Independent evolution of matrotrophy in the major classes of Bryozoa: transitions among reproductive patterns and their ecological background. Marine Ecology Progress Series 378:113–124.

Ostrovsky, A. N., A. V. Grischenko, P. D. Taylor, P. E. Bock, and S. F. Mawatari. 2006. Comparative anatomical study of internal brooding in three anascan bryozoans (Cheilostomata) and its taxonomic and evolutionary implications. Journal of Morphology 267:739–749.

Ostrovsky, A. N., P. Schäfer, and D. P. Gordon. 2003. Ultrastructure and development of the ooecial walls in some calloporid bryozoans (Gymnolaemata: Cheilostomata). Zoologischer Anzeiger 242:223–240.

Ostrovsky, A. N., and N. N. Shunatova. 2002. Colonial behaviour and group zooidal reactions in Bryozoa: history of research. Pages 185–199 *in* P. N. Wyse Jackson and M. E. Spencer Jones, editors. Annals of Bryozoology: Aspects of the History of Research on Bryozoans. International Bryozoology Association, Dublin.

Ostrovsky, A. N., N. N. Shunatova, and I. I. Antipenko. 2002. Historical review on individual autozooidal behaviour and feeding mechanisms in Bryozoa. Pages 201–227 *in* P. N. Wyse Jackson and M. E. Spencer Jones, editors. Annals of Bryozoology: Aspects of the History of Research on Bryozoans. International Bryozoology Association, Dublin.

Ostrovsky, A. N., and P. D. Taylor. 2004. Systematics of Upper Cretaceous calloporid bryozoans with primitive spinose ovicells. Palaeontology 47:775–793.

Ostrovsky, A. N., and P. D. Taylor. 2005. Brood chambers constructed from spines in fossil and Recent cheilostome bryozoans. Zoological Journal of the Linnean Society 144:317–361.

Ostrovsky, A. N., P. D. Taylor, M. H. Dick, and S. F. Mawatari. 2008a. Pre-Cenomanian cheilostome Bryozoa: current state of knowledge. Pages 69–74 *in* H. Okada, S. F. Mawatari, N. Suzuki, and P. Gautam, editors. Origin and Evolution of Natural Diversity. 21st Century COE for Neo-Science of Natural History, Hokkaido University.

Ostrovsky, A. N., N. Vávra, and J. S. Porter. 2008b. Sexual reproduction in gymnolaemate Bryozoa: History and perspectives of the research. *in* P. N. Wyse Jackson and M. E. Spencer Jones, editors. Annals of Bryozoology 2: Aspects of the History of Research on Bryozoans. International Bryozoology Association, Dublin.

O'Dea, A. 2006. Asexual propagation in the marine bryozoan *Cupuladria exfragminis*. Journal of Experimental Marine Biology and Ecology 335:312–322.

O'Dea, A. 2009. Relation of form to life habit in free-living cupuladriid bryozoans. Aquatic Biology 7:1–18.

O'Dea, A., E. Håkansson, P. D. Taylor, and B. Okamura. 2011. Environmental change prior to the K–T boundary inferred from temporal variation in the morphology of cheilostome bryozoans. Palaeogeography, Palaeoclimatology, Palaeoecology 308:502–512.

O'Dea, A., A. Herrera-Cubilla, H. Fortunato, and J. B. C. Jackson. 2004. Life history variation in cupuladriid bryozoans from either side of the Isthmus of Panama. Marine Ecology Progress Series 280:145–161.

O'Dea, A., and J. B. C. Jackson. 2002. Bryozoan growth mirrors contrasting seasonal regimes across the Isthmus of Panama. Palaeogeography, Palaeoclimatology, Palaeoecology 185:77–94.

O'Dea, A., J. B. C. Jackson, P. D. Taylor, and F. Rodríguez. 2008. Modes of reproduction in Recent and fossil cupuladriid bryozoans. Palaeontology 51:847–864.

O'Dea, A., and B. Okamura. 2000. Cheilostome bryozoans as indicators of seasonality in the Neogene epicontinental seas of western Europe. Pages 316–320 *in* A. Herrera Cubilla and J. B. C. Jackson, editors. Proceedings of the 11th International Bryozoology Association Conference. Smithsonian Tropical Research Institute, Balboa, Republic of Panama, Panama City.

O'Dea, A., A. N. Ostrovsky, and F. Rodríguez. 2010. Embryonic brooding and clonal propagation in tropical eastern Pacific cupuladriid bryozoans. Journal of the Marine Biological Association of the United Kingdom

90:291–299.

Pathmanaban, O. N., J. S. Porter, and I. R. White. 2005. Dogger Bank itch in the eastern English Channel: a newly described geographical distribution of an old problem. Clinical and Experimental Dermatology 30:622–626.

Perez, F. M., and W. C. Banta. 1996. How does *Cellaria* get out of its box? A new cheilostome hydrostatic mechanism (Bryozoa: Cheilostomata). Invertebrate Biology 115:162–169.

Pérez, L. M., M. Griffin, G. Pastorino, J. J. López Gappa, and M. O. Manceñido. 2015. Redescription and palaeoecological significance of the bryozoan *Hippoporidra patagonica* (Pallaroni, 1920) in the San Julián Formation (late Oligocene) of Santa Cruz province, Argentina. Alcheringa 39:1–7.

Pérez, L. M., J. J. López-Gappa, and M. Griffin. 2015. New and little-known bryozoans from Monte León Formation (early Miocene, Argentina) and their paleobiogeographic relationships. Journal of Paleontology 89:956–965.

Pirrie, D., J. A. Crame, J. B. Riding, A. R. Butcher, and P. D. Taylor. 1997. Miocene glaciomarine sedimentation in the northern Antarctic Peninsula region: the stratigraphy and sedimentology of the Hobbs Glacier Formation, James Ross Island. Geological Magazine 136:745–762.

Pizzaferri, C. 2010. New specimens of *Prenantia cheilostoma* (Manzoni) from the Pliocene of Castell'Arquato area (Western Emilia Region, N Italy). (Bryozoa Gymnolaemata Cheilostomata Smittinidae). Quaderno di Studi e Notizie di Storia Naturale della Romagna 31:89–117.

Pizzaferri, C., and B. Berning. 2007. Taxonomic notes on some cheilostome Bryozoa from the Pliocene of the Western Emilia region (N Italy). Rivista Italiana di Paleontologia e Stratigrafia 113:97–108.

Pizzaferri, C., and G. Braga. 2000. Nuove osservazioni sullo sviluppo astogenetico di Batopora rosula (Reuss), Bryozoa Cheilostomatida del Miocene del Pedeappennino parmense. Annali del Museo Civico di Rovereto 14:55–88.

Pohowsky, R. A. 1974. Notes on the study and nomenclature of boring Bryozoa. Journal of Paleontology 48:557–564.

Poignant, A. 1991. *Unifissurinella*, new genus of Microproblematica from the Upper Eocene of southwestern France. Micropaleontology 37:95–97.

Porter, J. S., J. S. Ryland, and G. R. Carvalho. 2002. Micro- and macrogeographic genetic structure in bryozoans with different larval strategies. Journal of Experimental Marine Biology and Ecology 272:119–130.

Powers, C. M., and J. F. Pachut. 2008. Diversity and distribution of Triassic bryozoans in the aftermath of the end-Permian mass extinction. Journal of Paleontology 82:362–371.

Pushkin, V. I., and L. E. Popov. 1999. Early Ordovician bryozoans from north-western Russia. Palaeontology 42:171–189.

Pushkin, V. I., and L. E. Popov. 2005. Two enigmatic bryozoans from the Middle Ordovician of the East Baltic. Palaeontology 48:1065–1074.

Ramalho, L. V., G. Muricy, and P. D. Taylor. 2009. Cyclostomata (Bryozoa, Stenolaemata) from Rio de Janeiro State, Brazil. Zootaxa 2057:32–52.

Reverter Gil, O., and E. Fernández Pulpeiro. 1995. *Chaperiopsis cristata* (Busk, 1884) (Bryozoa, Cheilostomata) is *Chaperiopsis annulus* (Manzoni, 1870). The occurrence of this species from the European Atlantic coast. Journal of Natural History 29:1057–1065.

Reverter Gil, O., and E. Fernández Pulpeiro. 2005. A new genus of cyclostome bryozoan from the European Atlantic coast. Journal of Natural History 39:2379–2387.

Reverter-Gil, O., and E. Fernández-Pulpeiro. 2007. Species of genus *Schizotheca* Hincks (Bryozoa, Cheilostomata) described in the Atlantic-Mediterranean region, with notes on some species of *Parasmittina* Osburn. Journal of Natural History 41:1929–1953.

Reverter-Gil, O., and J. Souto. 2015. Redescription of some species of Bryozoa described by J. Jullien and L. Calvet in the NE Atlantic. European Journal of Taxonomy 157:1–17.

Reverter-Gil, O., J. Souto, and E. Fernández Pulpeiro. 2009. Three new species of Iberian cheilostomate Bryozoa.

Journal of the Marine Biological Association of the United Kingdom 89:1499–1506.

Reverter-Gil, O., J. Souto, and E. Fernández-Pulpeiro. 2012. New and little known species of Bryozoa from Iberian Atlantic waters. Zoosystema 34:157–170.

Reverter-Gil, O., J. Souto, and E. Fernández-Pulpeiro. 2014. Annotated checklist of Recent marine Bryozoa from continental Portugal. Nova Acta Cientifica Compostelana (Bioloxía) 21:1–55.

Rogick, M. D. 1956. Studies on marine Bryozoa. VIII. *Exochella longirostris* Jullien 1888. Biological Bulletin 111:123–128.

Rogick, M. D. 1960. Studies on marine Bryozoa. XIII. Two new genera and new species from Antarctica. Biological Bulletin 119:479–493.

Ross, J. R. P. 1960. Larger cryptostome Bryozoa of the Ordovician and Silurian, Anticosti Island, Canada - Part I. Journal of Paleontology 34:1057–1076.

Ross, J. R. P. 1961. Larger cryptostome Bryozoa of the Ordovician and Silurian, Anticosti Island, Canada - Part II. Journal of Paleontology 35:331–344.

Ross, J. R. P. 1963. New Ordovician species of Chazyan trepostome and cryptostome Bryozoa. Journal of Paleontology 37:57–63.

Ross, J. R. P. 1964. Champlainian cryptostome Bryozoa from New York State. Journal of Paleontology 38:1–32.

Ross, J. R. P. 1967. Champlainian Ectoprocta (Bryozoa), New York State. Journal of Paleontology 41:632–648.

Ross, J. R. P. 1969. Champlainian (Ordovician) Ectoprocta (Bryozoa), New York State, Part II. Journal of Paleontology 43:257–284.

Ross, J. R. P. 1970. Distribution, paleoecology and correlation of Champlainian Ectoprocta (Bryozoa), New York State, Part III. Journal of Paleontology 44:346–382.

Rosso, A. 1999. Recent and fossil species of *Characodoma* Maplestone, 1900 (Bryozoa) from the Mediterranean with description of two new species. Journal of Natural History 33:415–437.

Rosso, A. 2002. *Amphiblestrum* Gray, 1848 (Bryozoa, Cheilostomata) from the Atlantic-Mediterranean area, with description of a new species. Journal of Natural History 36:1489–1508.

Rosso, A. 2003. Bryozoan diversity in the Mediterranean Sea. Biogeographia 24:227–250.

Rosso, A. 2004. Two new species of *Phylactella* (Bryozoa, Cheilostomatida) from the Mediterranean area belonging to the *P. labrosa* (Busk) complex of species. Journal of Natural History 38:2655–2668.

Rosso, A. 2008. Mediterranean setoselliniforms and their exploitation of small sized substrata. Pages 261–268 *in* S. J. Hageman, M. M. Key, and J. E. Winston, editors. Bryozoan studies 2007. Virginia Museum of Natural History, Martinsville, VA, USA.

Rosso, A. 2009. The first catenicellid (Bryozoa, Ascophora) from Mediterranean shallow waters: a hidden resident or a new immigrant? Journal of Natural History 43:2209–2226.

Rosso, A., and I. Di Geronimo. 1998. Deep-sea Pleistocene Bryozoa of southern Italy. Geobios 30:303–317.

Rosso, A., and M. Novosel. 2010. The genus Adeonella (Bryozoa, Ascophora) in the Mediterranean, with description of two new living species and rediscovery of a fossil one. Journal of Natural History 44:1697–1727.

Rosso, A., and R. Sanfilippo. 2005. Bryozoans and serpuloideans in skeletobiont communities from the Pleistocene of Siciliy: spatial utilisation and competitive interaction. Annali dell'Università degli Studi di Ferrara, Museologia Scientifica e Naturalistica, Volume speciale:17 pp.

Rosso, A., F. Sciuto, and A. Sinagra. 2010. Bertorsonidra n. gen. (Bryozoa, Cheilostomata) for *Tremoporaprenanti* Gautier, 1955, a rare species from the Mediterranean. Zoosystema 32:457–467.

Rosso, A., and P. D. Taylor. 2002. A new anascan cheilostome bryozoan from Icelandic deep waters and its uniserial colony growth pattern. Sarsia 87:35–46.

Rucker, J. B., and R. E. Carver. 1969. A survey of the carbonate mineralogy of cheilostome Bryozoa. Journal of Paleontology 43:791–799.

Ryland, J. S. 1969. A nomenclatural index to "A History of the British Marine Polyzoa" by T. Hincks (1880).

Bulletin of the British Museum (Natural History), Zoology 17:205–260.

Ryland, J. S. 1970. Bryozoans. Hutchinson University Library, London.

Ryland, J. S. 2001. Convergent colonial organization and reproductive function in two bryozoan species epizoic on gastropod shells. Journal of Natural History 35:1085–1101.

Ryland, J. S., J. D. D. Bishop, H. De Blauwe, A. El Nagar, D. Minchin, C. A. Wood, and A. L. E. Yunnie. 2011. Alien species of *Bugula* (Bryozoa) along the Atlantic coasts of Europe. Aquatic Invasions 6:17–31.

Ryland, J. S., H. De Blauwe, R. Lord, and J. A. Mackie. 2009. Recent discoveries of alien *Watersipora* (Bryozoa) in Western Europe, with redescriptions of species. Zootaxa 2093:43–59.

Ryland, J. S., and J. S. Porter. 2003. The identity of *Alcyonidium gelatinosum* (Linnaeus, 1761) (Bryozoa: Ctenostomatida). Journal of Natural History 37:2179–2189.

Sakagami, S. 1970. On the Paleozoic Bryozoa of Japan and Thai-Malayan districts. Journal of Paleontology 44:680–692.

Sakagami, S. 1999. Permian bryozoans from some localities in the Khao Hin Kling area near Phetchabun, north-central Thailand. Bulletin of the Kitakyushu Museum of Natural History 18:77–103.

Sakagami, S., D. Sciunnach, and E. Garzanti. 2006. Late Paleozoic and Triassic bryozoans from the Tethys Himalaya (N India, Nepal and S Tibet). Facies 52:279–298.

Sandberg, P. 1962. New cheilostome Bryozoa from the Miocene of the Dominican Republic. Micropaleontology 8:61–66.

Santagata, S., and W. C. Banta. 1996. Origin of brooding and ovicells in cheilostome bryozoans: interpretive morphology of Scrupocellaria ferox. Invertebrate Biology 115:170–180.

Santana, F. T., L. V. Ramalho, and C. P. Gumarães. 2009. A new species of *Metrarabdotos* (Bryozoa, Ascophora) from Brazil. Zootaxa 2222.

Schmid, H. P., M. Harzhauser, and A. Kroh. 2001. Hypoxic events on a Middle Miocene carbonate platform of the Central Paratethys (Austria, Badenian, 14 Ma). Annalen des Naturhistorischen Museums in Wien 102 A:1–50.

Schmidt, R. 2007. Australian Cenozoic Bryozoa, 2: free-living Cheilostomata of the Eocene St. Vincent Basin, S.A., including Bonellina gen. nov. Alcheringa 31:67–84.

Schmidt, R., and Y. Bone. 2003. Biogeography of Eocene bryozoans from the St. Vincent Basin, South Australia. Lethaia 36:345–356.

Schneider, S., B. Berning, M. A. Bitner, R.-P. Carriol, M. Jäger, J. Kriwet, A. Kroh, and W. Werner. 2009. A parautochthonous shallow marine fauna from the Late Burdigalian (early Ottnangian) of Gurlarn (Lower Bavaria, SE Germany): macrofaunal inventory and paleoecology. Neues Jahrbuch für Geologie und Paläontologie, Abhandlungen 254:63–103.

Schopf, T. J. M. 1969. Paleoecology of ectoprocts (bryozoans). Journal of Paleontology 43:234–244.

Schopf, T. J. M., and F. T. Manheim. 1967. Chemical composition of Ectoprocta (Bryozoa). Journal of Paleontology 41:1197–1225.

Sendino, C., and P. D. Taylor. 2011. Sir Charles Lyell's fossil bryozoans from Gran Canaria. Pages 123–142 *in* P. N. Wyse Jackson and M. E. Spencer Jones, editors. Annals of Bryozoology 3: Aspects of the History of Research on Bryozoans. International Bryozoology Association, Dublin.

Shapiro, D. F. 1996. Size-dependent neural integration between genetically different colonies of a marine bryozoan. The Journal of Experimental Biology 199:1229–1239.

Shier, D. E. 1964. Marine Bryozoa from northwest Florida. Bulletin of Marine Science of the Gulf and Caribbean 14:603–662.

Shunatova, N. N., and A. N. Ostrovsky. 2002. Group autozooidal behaviour and chimneys in marine bryozoans. Marine Biology 140:503–518.

Smith, A. M. 1995. Palaeoenvironmental interpretation using bryozoans: a review. Pages 231–243 *in* D. W. J. Bosence and P. A. Allison, editors. Marine Palaeoenvironmental Analysis from Fossils. Geological Society Special Publication 83.

Smith, A. M. 2014. Growth and calcification of marine bryozoans in a changing ocean. Biological Bulletin 226:203–210.

Smith, A. M., and E. Girvan. 2010. Understanding a bimineralic bryozoan: Skeletal structure and carbonate mineralogy of *Odontionella cyclops* (Foveolariidae: Cheilostomata: Bryozoa) in New Zealand. Palaeogeography, Palaeoclimatology, Palaeoecology 289:113–122.

Smith, A. M., M. M. Key, and D. P. Gordon. 2006. Skeletal mineralogy of bryozoans: taxonomic and temporal patterns. Earth-Science Reviews 78:287–306.

Smith, A. M., B. Stewart, M. M. Key, and C. M. Jamet. 2001. Growth and carbonate production by *Adeonellopsis* (Bryozoa: Cheilostomata) in Doubtful Sound, New Zealand. Palaeogeography, Palaeoclimatology, Palaeoecology 175:201–210.

Sogot, C. E., E. M. Harper, and P. D. Taylor. 2014. The Lilliput effect in colonial organisms: cheilostome bryozoans at the Cretaceous–Paleogene mass extinction. Public Library of Science: ONE 9:e87048.

Sonar, M. A., and R. M. Badve. 2008. Fossil cyclostome bryozoans from the Holocene rocks of west coast of Maharashtra and Goa, India. Gondwana Geological Magazine 23:69–76.

Sonar, M. A., R. M. Badve, and S. G. Gaikwad. 2010. Usefulness of Bryozoa to deduce palaeoenvironment: a case study from the Holocene of west coast of Maharashtra and Goa. Gondwana Geological Magazine 25:69–80.

Sonar, M. A., and S. G. Gaikwad. 2013a. Cyclostome Bryozoa from the Cenozoic sediments of western Kachchh, Gujarat, India. Journal of the Palaeontological Society of India 58:195–203.

Sonar, M. A., and S. G. Gaikwad. 2013b. Fossil neocheilostomine bryozoans from the Tertiary rocks of Western Kachchh, Gujarat. Journal of the Geological Society of India 81:665–676.

Sonar, M. A., and S. G. Gaikwad. 2013c. Fossil steginoporellid (Cheilostomata: Neocheilostomina), Bryozoa from the Tertiary sediments of Western Kachchh, Gujarat, India. Journal of Earth System Science 122:149–161.

Souaya, F. J. 1965. On the Bryozoa of Gebel Gharra (Cairo-Suez Road) and other Miocene sections in Egypt. Journal of Paleontology 39:1129–1144.

Soule, J. D. 1973. Histological and histochemical studies on the bryozoan-substrate interface. Pages 343–347 *in* G. P. Larwood, editor. Living and Fossil Bryozoa. Academic Press, London.

Soule, J. D., and D. F. Soule. 1969. Systematics and biogeography of burrowing bryozoans. American Zoologist 9:791–802.

Soule, J. D., and D. F. Soule. 1970. New species of *Thalamoporella* (Ectoprocta) from Hawaii, examined by scanning electron microscopy. American Museum Novitates 2417:1–18.

Souto, J., O. Reverter-Gil, and E. Fernández-Pulpeiro. 2010. Bryozoa from detritic bottoms in the Menorca Channel (Balearic Islands, western Mediterranean), with notes on the genus *Cribellopora.* Zootaxa 2536:36–52.

Souto, J., O. Reverter-Gil, and E. Fernández-Pulpeiro. 2011. Redescription of some bryozoan species originally described by J. Jullien from Iberian waters. Zootaxa 2827:31–53.

Souto, J., O. Reverter-Gil, and A. N. Ostrovsky. 2014. New species of Bryozoa from Madeira associated with rhodoliths. Zootaxa 3795:135–151.

Stach, L. W. 1936. Studies on Recent Petraliidae (Bryozoa). Records of the Australian Museum 19:355–379.

Steinthorsdottir, M., S. Lidgard, and E. Håkansson. 2006. Fossils, sediments, tectonics. Reconstructing palaeoenvironments in a Pliocene–Pleistocene Mediterranean microbasin. Facies 52:361–380.

Suárez-Andrés, J. L., and A. Ernst. 2015. Lower–Middle Devonian Fenestellidae (Bryozoa) of NW Spain: implications for fenestrate palaeobiogeography. Facies 61:415.

Suárez-Andrés, J. L., and P. N. Wyse Jackson. 2014. *Ernstipora mackinneyi*, a new unique fenestrate bryozoan genus and species with an encrusting growth habit from the Emsian (Devonian) of NW Spain. Neues Jahrbuch für Geologie und Paläontologie, Abhandlungen 271:229–242.

Suárez-Andrés, J. L., and P. N. Wyse Jackson. 2015. Feeding currents: a limiting factor for disparity of Palaeozoic fenestrate bryozoans. Palaeogeography, Palaeoclimatology, Palaeoecology 433:219–232.

Suttner, T. J., and A. Ernst. 2007. Upper Ordovician bryozoans of the Pin Formation (Spiti Valley, northern

India). Palaeontology 50:1485–1518.

Szczechura, J. 1994. A segmented Paleocene cheilostomatous bryozoan and its possible relationships with pseudarcellids. Acta Palaeontologica Polonica 39:223–231.

Sørensen, A. M., E. Håkansson, and L. Stemmerik. 2007. Faunal migration into the Late Permian Zechstein Basin – evidence from bryozoan palaeobiogeography. Palaeogeography, Palaeoclimatology, Palaeoecology 251:198–209.

Tang, S., and R. J. Cuffey. 1998. *Inconobotopora lichenoporoides* a new genus and species of cystoporate bryozoan from the Silurian of Gotland, and its evolutionary implication. Journal of Paleontology 72:2562–64.

Taylor, P. D. 1978. The spiral bryozoan *Terebellaria* from the Jurassic of southern England and Normandy. Palaeontology 21:357–391.

Taylor, P. D. 1982. Probable predatory borings in Late Cretaceous bryozoans. Lethaia 15:67–74.

Taylor, P. D. 1986. The ancestrula and early growth pattern in two primitive cheilostome bryozoans: *Pyripora catenularia* (Fleming) and *Pyriporopsis portlandensis* Pohowsky. Journal of Natural History 20:101–110.

Taylor, P. D. 2001. Preliminary systematics and diversity patterns of cyclostome bryozoans from the Neogene of the Central American Isthmus. Journal of Paleontology 75:578–589.

Taylor, P. D. 2008. Late Cretaceous cheilostome bryozoans from California and Baja California. Journal of Paleontology 82:823–834.

Taylor, P. D. 2009. Bryozoans from the Middle Jurassic of Balin, Poland: a revision of material described by A.E. Reuss (1867). Annalen des Naturhistorischen Museums in Wien 110A:17–54.

Taylor, P. D., B. Berning, and M. A. Wilson. 2013. Reinterpretation of the Cambrian 'bryozoan' *Pywackia* as an octocoral. Journal of Paleontology 87:984–990.

Taylor, P. D., and P. L. Cook. 1981. *Hippoporidra edax* (Busk 1859) and a revision of some fossil and living Hippoporidra (Bryozoa). Bulletin of the British Museum (Natural History), Geology 35:243–251.

Taylor, P. D., and E. Di Martino. 2014. Why is the tropical Cenozoic fossil record so poor for bryozoans? Studi Trentini di Scienze Naturali 94:249–257.

Taylor, P. D., M. H. Dick, D. Clements, and S. F. Mawatari. 2012. A diverse bryozoan fauna from Pleistocene marine gravels at Kuromatsunai, Hokkaido, Japan. Pages 367–383 *in* A. Ernst, P. Schäfer, and J. Scholz, editors. Bryozoan Studies 2010. Springer, Berlin.

Taylor, P. D., and A. Ernst. 2008. Bryozoans in transition: the depauperate and patchy Jurassic biota. Palaeogeography, Palaeoclimatology, Palaeoecology 263:9–23.

Taylor, P. D., and D. P. Gordon. 1997. *Fenestulipora*, gen. nov., an unusual cyclostome bryozoan from New Zealand and Indonesia. Invertebrate Taxonomy 11:689–703.

Taylor, P. D., and D. P. Gordon. 2002. Alcide d'Orbigny's work on Recent and fossil bryozoans. Comptes Rendus Palevol 1:533–547.

Taylor, P. D., and D. P. Gordon. 2003. Endemic new cyclostome bryozoans from Spirits Bay, a New Zealand marine-biodiversity "hotspot". New Zealand Journal of Marine and Freshwater Research 37:653–669.

Taylor, P. D., D. P. Gordon, and P. B. Batson. 2004. Bathymetric distributions of modern populations of some common Cenozoic Bryozoa from New Zealand, and paleodepth estimation. New Zealand Journal of Geology and Geophysics 47:57–69.

Taylor, P. D., and N. P. James. 2013. Secular changes in colony-forms and bryozoan carbonate sediments through geological history. Sedimentology 60:1184–1212.

Taylor, P. D., N. P. James, Y. Bone, P. Kuklinski, and T. K. Kyser. 2009a. Evolving mineralogy of cheilostome bryozoans. Palaios 24:440–452.

Taylor, P. D., P. Kuklinski, and D. P. Gordon. 2007. Branch diameter and depositional depth in cyclostome bryozoans: testing a potential paleobathymetric tool. Palaios 22:220–224.

Taylor, P. D., D. G. Lazo, and M. B. Aguirre-Ureta. 2009b. Lower Cretaceous bryozoans from Argentina: a 'by-catch' fauna from the Agrio Formation (Neuquén Basin). Cretaceous Research 30:193–203.

Taylor, P. D., and F. K. McKinney. 1996. An Archimedes-like cyclostome bryozoan from the Eocene of North

Carolina. Journal of Paleontology 70:218–229.

Taylor, P. D., and F. K. McKinney. 2000. Reinterpretation of *Stictostega* Shaw, 1967, an Upper Cretaceous cheilostome bryozoan from Arkansas. Journal of Paleontology 74:1–6.

Taylor, P. D., and K. S. Schindler. 2004. A new Eocene species of the hermit-crab symbiont *Hippoporidra* (Bryozoa) from the Ocala Limestone of Florida. Journal of Paleontology 78:790–794.

Taylor, P. D., and C. Sendino. 2010. Latitudinal distribution of bryozoan-rich sediments in the Ordovician. Bulletin of Geosciences 85:565–572.

Taylor, P. D., O. Vinn, and M. A. Wilson. 2010. Evolution of biomineralization in "lophophorates". Palaeontology 84:317–333.

Taylor, P. D., and A. Waeschenbach. 2015. Phylogeny and diversification of bryozoans. Palaeontology 58:585–599.

Taylor, P. D., A. Waeschenbach, and W. K. Florence. 2011. Phylogenetic position and systematics of the bryozoan *Tennysonia*: further evidence for convergence and plasticity in skeletal morphology among cyclostome bryozoans. Zootaxa 3010:58–68.

Taylor, P. D., and M. A. Wilson. 2002. A new terminology for marine organisms inhabiting hard substrates. Palaios 17:522–525.

Taylor, P. D., and M. A. Wilson. 2003. Palaeoecology and evolution of marine hard substrate communities. Earth-Science Reviews 62:1–103.

Taylor, P. D., M. A. Wilson, and R. G. Bromley. 1999. A new ichnogenus for etchings made by cheilostome bryozoans into calcareous substrates. Palaeontology 42:595–604.

Taylor, P. D., and P. M. Zaborski. 2002. A Late Cenomanian bryozoan biostrome from north-eastern Nigeria. Cretaceous Research 23:241–253.

Taylor, P. D., and M. Zaton. 2008. Taxonomy of the bryozoan genera *Oncousoecia, Microeciella and Eurystrotos* (Cyclostomata: Oncousoeciidae). Journal of Natural History 42:2557–2574.

Taylor, P. D., and K. Zágorsek. 2011. Operculate cyclostome bryozoans (Eleidae) from the Bohemian Cretaceous. Paläontologische Zeitschrift 85:407–432.

Tenison-Woods, J. E. 1880. Palaeontology of New Zealand. Part IV. Corals and Bryozoa of the Neozoic Period in New Zealand. Page 34. G. Didsbury, Government Printer, Wellington.

Thomson, W. T. C. 1852. Notes on some Scotch Zoophytes and Polyzoa. Annals and Magazine of Natural History, series 2 9:403–404.

Tilbrook, K. J. 2001. Indo-West Pacific species of the genus *Stylopoma* Levinsen, 1909 (Bryozoa: Cheilostomatida). Zoological Journal of the Linnean Society 131:1–34.

Tilbrook, K. J. 2011. New genus for a unique species of Indo-West Pacific bryozoan. Zootaxa 3134:63–67.

Tilbrook, K. J., and D. P. Gordon. 2015. Bryozoa from the Straits of Johor, Singapore, with the description of a new species. Raffles Bulletin of Zoology 31:255–263.

Tilbrook, K. J., and A. V. Grischenko. 2004. New sub-Arctic species of the tropical genus *Antropora* (Bryozoa: Cheilostomata): a gastropod-pagurid crab associate. Journal of the Marine Biological Association of the United Kingdom 84:1001–1004.

Tilbrook, K. J., P. J. Hayward, and D. P. Gordon. 2001. Cheilostomatous Bryozoa from Vanuatu. Zoological Journal of the Linnean Society 131:35–109.

Tolokonnikova, Z. A., and A. Ernst. 2010. Palaeobiogeography of Famennian (Late Devonian) bryozoans. Palaeogeography, Palaeoclimatology, Palaeoecology 298:360–369.

Tompsett, S., J. S. Porter, and P. D. Taylor. 2009. Taxonomy of the fouling cheilostome bryozoans *Schizoporella unicornis* (Johnston) and *Schizoporella errata* (Waters). Journal of Natural History 43:2227–2243.

Toots, H., and J. F. Cutler. 1962. Bryozoa from the "Mesaverde" Formation (Upper Cretaceous) of southeastern Wyoming. Journal of Paleontology 36:81–86.

Toscano, F., and P. D. Taylor. 2008. A new Early Miocene bryozoan, *Favosipora ichnusae* sp. nov. (Cyclostomata), from the Isili Limestone of Sardinia, Italy. Neues Jahrbuch für Geologie und Paläontologie Abhandlungen

248:301–308.

Tuckey, M. E. 1990. Distributions and extinctions of Silurian Bryozoa. Pages 197–206 *in* W. S. McKerrow and C. R. Scotese, editors. Palaeozoic Palaeogeography and Biogeography. Geological Society, London.

Urbanek, A. 2004. Morphogenetic gradients in graptolites and bryozoans. Acta Palaeontologica Polonica 49:485–504.

Utgaard, J. 1981. *Lunaferamita*, a new genus of Constellariidae (Bryozoa) with strong cystoporate affinities. Journal of Paleontology 55:1058–1070.

Vávra, N. 1980. Tropische Faunenelemente in den Bryozoenfaunen des Badenien (Mittelmiozän) der Zentralen Paratethys. Sitzungsberichte der Österreichischen Akademie der Wissenschaften, Mathematisch-naturwissenschaftliche Klasse, Abteilung 1 189:49–63.

Vieira, L. M., and D. P. Gordon. 2010. *Eutaleola*, a replacement name for the homonym *Euteleia* (Bryozoa: Pasytheidae). Zoologia 27:646–648.

Vieira, L. M., D. P. Gordon, and M. D. Correia. 2007. First record of a living ditaxiporine catenicellid in the Atlantic, with a description of *Vasignyella ovicellata* n. sp. (Bryozoa). Zootaxa 1582:49–58.

Vieira, L. M., D. P. Gordon, F. B. C. Souza, and M. A. Haddad. 2010. New and little-known cheilostomatous Bryozoa from the south and southeastern Brazilian continental shelf and slope. Zootaxa 2722:1–53.

Vieira, L. M., M. E. Spencer Jones, and J. E. Winston. 2013. *Cradoscrupocellaria*, a new bryozoan genus for *Scrupocellaria bertholletii* (Audouin) and related species (Cheilostomata, Candidae): taxonomy, biodiversity and distribution. Zootaxa 3707:1–63.

Vieira, L. M., M. E. Spencer Jones, J. E. Winston, A. E. Migotto, and A. C. Marques. 2014. Evidence for polyphyly of the genus *Scrupocellaria* (Bryozoa: Candidae) based on a phylogenetic analysis of morphological characters. Public Library of Science: ONE 9:e95296.

Vieira, L. M., and S. N. Stampar. 2014. A new *Fenestrulina* (Bryozoa, Cheilostomata) commensal with tube-dwelling anemones (Cnidaria, Ceriantharia) in the tropical southwestern Atlantic. Zootaxa 3780:365–374.

Vine, G. R. 1884. Polyzoa (bryozoa) found in the boring at richmond, surrey referred to by prof. Judd, frs. Quarterly Journal of the Geological Society 40:784–794.

Viskova, L. A., and A. V. Koromyslova. 2012. *Tamanicella* gen. nov., a new genus of bryozoans forming the Late Miocene bioherms of Cape Panagia in the Taman Peninsula (Russia). Paleontological Journal 46:29–43.

Voigt, E. 1964. A bryozoan fauna of Dano-Montian age from Boryszew and Sochaczew in Central Poland. Acta Palaeontologica Polonica 9:419–498.

Voigt, E. 1968. On the Cretaceous age of the so-called Jurassic cheilostomatous Polyzoa (Bryozoa). A contribution to the knowledge of the Polyzoa-fauna of the Maastrichtian in the Cotentin (Manche). Bulletin of the British Museum (Natural History), Geology 17:1–45.

Waeschenbach, A., J. S. Porter, and R. N. Hughes. 2012. Molecular variability in the *Celleporella hyalina* (Bryozoa; Cheilostomata) species complex: evidence for cryptic speciation from complete mitochondrial genomes. Molecular Biology Reports 39:8601–8614.

Wass, R. E. 1972. Permian Bryozoa from South Africa. Journal of Paleontology 46:871–873.

Wass, R. E. 1977. Branching patterns and phylogeny of the family Vittaticellidae (Bryozoa: Cheilostomata). Australian Journal of Zoology 25:103–119.

Wass, R. E., and J. J. Yoo. 1975. Bryozoa from Site 282 West Tasmania. Pages 809–831 *in* J. P. Lemmet, R. E. Houtz, and E. al., editors. Initial Reports of the Deep Sea Drilling Project, Volume 29. U.S Government Printing Office, Washington, USA.

Wass, R. E., and J. J. Yoo. 1983. Cheilostome Bryozoa from the southern Australian continental shelf. Australian Journal of Marine and Freshwater Research 34:303–354.

Waters, A. W. 1881. On fossil chilostomatous Bryozoa from south-west Victoria, Australia. Quarterly Journal of the Geological Society of London 37:309–347.

Waters, A. W. 1882. On chilostomatous Bryozoa from Bairnsdale (Gippsland). Quarterly Journal of the Geological

Society of London 38:502–513.

Waters, A. W. 1883. On fossil chilostomatous Bryozoa from Muddy Creek, Victoria. Quarterly Journal of the Geological Society of London 39:423–443.

Waters, A. W. 1884a. Fossil cyclostomatous Bryozoa from Australia. Quarterly Journal of the Geological Society of London 40:674–697.

Waters, A. W. 1884b. Closure of the cyclostomatous Bryozoa. Journal of the Linnean Society of London, Zoology 17:400–404.

Waters, A. W. 1885. Chilostomatous Bryozoa from Aldinga and the River-Murray Cliffs, South Australia. Quarterly Journal of the Geological Society of London 41:279–310.

Waters, A. W. 1888. Supplementary Report on the Polyzoa collected by H.M.S. 'Challenger' during the years 1873-1876. Report on the Scientific Results of the Voyage of the H.M.S. 'Challenger'. Zoology Part LXXIX 31:1–41.

Waters, A. W. 1889. Bryozoa from New South Wales. Part IV. Annals and Magazine of Natural History, series 6 4:1–24.

Waters, A. W. 1898. Observations on Membraniporidae. Journal of the Linnean Society (Zoology) 26:654–693.

Waters, A. W. 1904a. Bryozoa. Résultats du Voyage du S.Y. Belgica en 1897-1898-1899, Zoologie 4:1–114.

Waters, A. W. 1904b. Bryozoa from Franz-Josef Land, collected by the Jackson-Harmsworth expedition, 1896-1897. Part 2, Cyclostomata, Ctenostomata, and Entoprocta. Journal of the Linnean Society (Zoology) 29:161–184.

Waters, A. W. 1905. Bryozoa from near Cape Horn. Journal of the Linnean Society (Zoology) 29:230–251.

Waters, A. W. 1907. *Tubucellaria*: its species and ovicells. Journal of the Linnean Society (Zoology) 30:126–133.

Weedon, M. J., and P. D. Taylor. 1995. Calcitic nacreous ultrastructures in bryozoans: implications for comparative biomineralization of lophophorates and molluscs. Biological Bulletin 188:281–292.

Weedon, M. J., and P. D. Taylor. 1996. Skeletal ultrastructures in some cerioporine cyclostome bryozoans. Acta Zoologica 77:249–265.

West, R. R., F. K. McKinney, J. A. Fagerstrom, and J. Vacelet. 2011. Biological interactions among extant and fossil clonal organisms. Facies 57:351–374.

Williams, J. D., and J. J. McDermott. 2004. Hermit crab biocoenoses: a worldwide review of the diversity and natural history of hermit crab associates. Journal of Experimental Marine Biology and Ecology 305:1–128.

Williams, M., A. M. Haywood, E. M. Harper, A. L. A. Johnson, T. Knowles, M. J. Leng, D. J. Lunt, B. Okamura, P. D. Taylor, and J. Zalasiewicz. 2009. Pliocene climate and seasonality in North Atlantic shelf seas. Philosophical Transactions of the Royal Society of London, Ser. A 367:85–108.

Wilson, M. A., and P. D. Taylor. 2001. Palaeoecology of hard substrate faunas from the Cretaceous Qahlah Formation of the Oman Mountains. Palaeontology 44:21–41.

Wilson, M. A., and P. D. Taylor. 2006. Predatory drill holes and partial mortality in Devonian colonial metazoans. Geology 34:565–568.

Winston, J. E. 1977. Feeding in marine bryozoans. Pages 233–271 *in* R. M. Woollacott and R. L. Zimmer, editors. Biology of Bryozoans. Academic Press, New York.

Winston, J. E., and S. E. Beaulieu. 1999. *Striatodoma dorothea* (Cheilostomatida: Tessaradomidae), a new genus and species of bryozoan from deep water off California. Proceedings of the Biological Society of Washington 112:313–318.

Winston, J. E., and B. F. Heimberg. 1986. Bryozoans from Bali, Lombok, and Komodo. American Museum Novitates 2847:1–49.

Winston, J. E., and J. B. C. Jackson. 1984. Ecology of cryptic coral reef communities. IV. Community development and life histories of encrusting cheilostome Bryozoa. Journal of Experimental Marine Biology and Ecology 76:1–21.

Winston, J. E., and L. M. Vieira. 2013. Systematics of interstitial encrusting bryozoans from southeastern Brazil. Zootaxa 3710:101–146.

Winston, J. E., L. M. Vieira, and R. M. Woollacott. 2014. Scientifi results of the Hassler Expedition. Bryozoa No. 2. Brazil. Bulletin of the Museum of Comparative Zoology 161:139–239.

Winston, J. E., and R. M. Woollacott. 2009. Scientific results of the Hassler Expedition. Bryozoa. No. 1 Barbados. Bulletin of the Museum of Comparative Zoology 159:239–300.

Wood, A. C. L., P. K. Probert, A. A. Rowden, and A. M. Smith. 2012. Complex habitat generated by marine bryozoans: a review of its distribution, structure, diversity, threats and conservation. Aquatic Conservation: Marine and Freshwater Ecosystems 22:547–563.

Wyse Jackson, P. N., and C. J. Buttler. 2015. Part G, Revised, Volume 2, Chapter 3: Preparation, imaging, and conservation of Paleozoic bryozoans for study. Treatise Online 63:1–15.

Wyse Jackson, P. N., E. Cross, and R. Swift. 2009. *Ptilofenestella*, a distinctive rare fenestrate bryozoan from the Mississippian of Ireland: new records and an extension of its stratigraphical range. Irish Naturalist's Journal 30:76–77.

Wyse Jackson, P. N., and M. M. Key. 2007. Borings in trepostome bryozoans from the Ordovician of Estonia: two ichnogenera produced by a single maker, a case of host morphology control. Lethaia 40:237–252.

Wyse Jackson, P. N., and M. M. Key. 2014. Epizoic bryozoans on cephalopods through the Phanerozoic: a review. Studi Trentini di Scienze Naturali 94:283–291.

Wyse Jackson, P. N., and F. K. McKinney. 2013. A micro-computed tomography and scanning electron microscopy investigation of the structure of *Polyfenestella* Bancroft, 1986 (Bryozoa: Fenestrata), from the Mississippian of Scotland: revealing the natire of its heteromorphs. Irish Journal of Earth Sciences 31:1–6.

Wyse Jackson, P. N., F. K. McKinney, and A. Ernst. 2010. Retepora undata M'Coy, 1844, type species of *Phylloporella* Frederiks, 1916, a junior synonym of Chainodictyon Foerste, 1877: a phylloporinid bryozoan from the Mississippian of Ireland redescribed. Irish Journal of Earth Sciences 28:53–60.

Wyse Jackson, P. N., C. M. Reid, and F. K. McKinney. 2011. The status of Protoretepora de Koninck, 1878 (Fenestrata: Bryozoa), and description of *P. crockfordae* sp. nov. and *P. wassi* sp. nov. from the Permian of Australia. Alcheringa 35:539–552.

Wyse Jackson, P. N., C. M. Reid, and F. K. McKinney. 2012. Fixation of the type species of the genus *Protoretepora* de Koninck, 1878 (Bryozoa, Fenestrata). Alcheringa: An Australasian Journal of Palaeontology 36:137–138.

Xia, F.-S., S.-G. Zhang, and Z.-Z. Wang. 2007. The oldest bryozoans: new evidence from the Late Tremadocian (Early Ordovician) of East Yangtze Gorges in China. Journal of Paleontology 81:1308–1326.

Yagunova, E. B., and A. N. Ostrovsky. 2008. Encrusting bryozoan colonies on stones and algae: variability of zooidal size and its posssible causes. Journal of the Marine Biological Association of the United Kingdom 88:901–908.

Zabala, M., and P. Maluquer. 1988. Illustrated Keys for the Classification of Mediterranean Bryozoa. Ajuntament de Barcelona, Barcelona.

Zaton, M., and P. D. Taylor. 2009. Middle Jurassic cyclostome bryozoans from the Polish Jura. Acta Palaeontologica Polonica 54:267–288.

Zágorsek, K. 2003. Upper Eocene Bryozoa from Waschberg Zone (Austria). Beiträge zur Paläontologie 28:101–263.

Zágorsek, K. 2010. Bryozoa from the Langhian (Miocene) of the Czech Republic. Part II: Systematic description of the suborder Ascophora Levinsen, 1909 and paleoecological reconstruction of the studied paleoenvironment. Acta Musei Nationalis Pragae, Series B - Historia Naturalis 66:139–255.

Zágorsek, K., S. Filipescu, and K. Holcová. 2010. New Middle Miocene Bryozoa from Gârbova de Sus (Romania) and their relationship to the sedimentary environment. Geologica Carpathica 61:495–512.

Zágorsek, K., and K. Fordinál. 2006. Lower Sarmatian Bryozoa from brackish sediment in the northern part of the Danube Basin (Dubová, Slovakia). Linzer biologische Beiträge 38:93–99.

Zágorsek, K., and D. P. Gordon. 2013. Late Tortonian bryozoans from Mut Basin, Central Anatolian Plateau, southern Turkey. Acta Palaeontologica Polonica 58:595–607.

Zágorsek, K., and D. P. Gordon. 2014. Revision of the Oligocene bryozoan taxa described by Stoliczka (1862), with the description of a new genus of Bryocryptellidae. Geodiversitas 36:541–564.

Zágorsek, K., K. Holcová, S. Nehyba, A. Kroh, and S. Hladilová. 2009. The invertebrate fauna of the Middle Miocene (lower Badenian) sediments of Kralice nad Oslavou (Central Paratethys, Moravian part of the Carpathian Foredeep). Bulletin of Geosciences 84:465–496.

Zágorsek, K., A. N. Ostrovsky, and N. Vávra. 2011. The new cheilostome bryozoan *Metrarabdotos nehybai* from the Middle Miocene of Moravia (Czech Republic): palaeofaunistic, taxonomic and ontogenetic aspects. Neues Jahrbuch für Geologie und Paläontologie, Abhandlungen 260:21–31.

Zágorsek, K., U. Radwanska, and A. Radwanski. 2012. Bryozoa from the Korytnica Basin (Middle Miocene; Holy Cross Mountains, Central Poland). Bulletin of Geosciences 87:201–218.

Zágorsek, K., L. V. Ramalho, B. Berning, and V. A. Távora. 2014. A new genus of the family Jaculinidae (Cheilostomata, Bryozoa) from the Miocene of the tropical western Atlantic. Zootaxa 3838:98–112.

Zágorsek, K., L. Silye, and B. Szabó. 2008. New Bryozoa from the Sarmatian (Middle Miocene) deposits of the Cerna-Strei Depression Romania. Studia Universitatis Babes-Bolyai, Geologia 53:25–29.

Zágorsek, K., N. Vávra, and K. Holcová. 2007. New and unusual Bryozoa from the Badenian (Middle Miocene) of the Moravian part of the Vienna Basin (Central Paratethys, Czech Republic). Neues Jahrbuch für Geologie und Paläontologie, Abhandlungen 243:201–215.

Zágoršek, K., D. P. Gordon, and N. Vávra. 2015. Revision of Chlidoniopsidae Harmer, 1957 (Bryozoa: Cheilostomata) including a description of *Celiopsis vici* gen. and sp. nov. Journal of Paleontology 89:140–147.

Zenetos, A., M. E. Çinar, M. A. Pancucci-Papadopoulou, J.-G. Harmelin, G. Furnari, F. Andaloro., N. Bellou, N. Streftaris, and H. Zibrowius. 2005. Annotated list of marine alien species in the Mediterranean with records of the worst invasive species. Mediterranean Marine Science 6:63–118.

Zenetos, A., S. Gofas, C. Morri, A. Rosso, and et Al. 2012. Alien species in the Mediterranean Sea by 2012. A contribution to the application of European Union's Marine Strategy Framework Directive (MSFD). Part 2. Introduction trends and pathways. Mediterranean Marine Science 13:328–352.

Zhang, S.-G., F.-S. Xia, H.-J. Yan, and Z.-Z. Wang. 2009. Horizon of the oldest known bryozoans (Ordovician). Palaeoworld 18:67–73.

Ziko, A., Y. A. El Safori, A. El Sorogy, M. Abd El-Wahab, N. El Dera, and W. Sheata. 2012. Bryozoa from northern Red Sea, Egypt: 1 *Crisia* (Cyclostomata). Historical Biology 24:113–119.
